# When AI chatbots understand emotions: exploring the mechanisms of self-disclosure from emotional arousal to psychological acceptance—a hybrid SEM–ANN–NCA analysis across multiple interaction configurations

**DOI:** 10.3389/fpsyg.2025.1724313

**Published:** 2026-02-19

**Authors:** Xiaojie Peng, Yanran Qian, Yuanyuan He

**Affiliations:** 1 College of Art and Design, Wuhan Textile University, Wuhan, China; 2 Major of Design, Department of Design, Graduate School, Hanyang University, Seoul, Republic of Korea

**Keywords:** AI chatbots, ANN, interaction modes, NCA, PLS-SEM, self-disclosure, S–O–R model

## Abstract

Emotions play a central role in shaping individuals’ cognitive responses and selfexpression, while AI chatbots are emerging as novel mediators for psychological support and self-exploration. However, existing research has often simplified human–machine interactions into linear cognitive processes, overlooking the underlying nonlinear and necessary psychological mechanisms. This study constructs a multilayered model integrating emotional arousal, affective engagement, and psychological acceptance to reveal how different interaction modes (text, voice, and multimodal) influence users’ self-disclosure behaviors. Based on 352 valid survey responses, the study employed Structural Equation Modeling (SEM) to validate the core path relationships and further integrated Artificial Neural Network (ANN) and Necessary Condition Analysis (NCA) to uncover nonlinear effects and necessity thresholds among latent variables. Results indicated that voice and multimodal interactions significantly enhanced users’ emotional arousal and psychological acceptance, both of which served as key mechanisms facilitating self-disclosure. Moreover, the ANN analysis revealed the non-compensatory nature of interaction mode effects, while the NCA results further identified emotional arousal and acceptance as indispensable conditions for high levels of self-disclosure. These findings suggest that AI chatbots should not be viewed merely as tools for information exchange but rather as co-constructs of emotion and trust, whose interaction design should focus on the dynamic balance between affective engagement and psychological safety. This study provides a novel theoretical perspective for understanding human–AI emotional resonance and psychological expression, as well as design implications for developing AI intervention systems with enhanced psychological sensitivity.

## 1 Introduction

In recent years, mental health issues among university students have increasingly intensified, with anxiety, depression, and stress overload emerging as major impediments to academic achievement and overall wellbeing [World Health Organization (WHO), 2022]. At the same time, the emergence of digital psychological intervention tools has been recognized as a complementary advancement to traditional counseling paradigms (Andersson, 2018). According to prior research, the evolution of mental health interventions can be delineated into four progressive stages: early self-help manuals, web-based platforms, mobile applications, and, most recently, AI-driven intelligent agents (Torous et al., 2021). Among these, AI chatbots—distinguished by their anonymity, accessibility, and immediacy—are regarded as the latest generation of digital intervention modalities and have increasingly been classified within academic discourse as “digital mental health agents” (Inkster et al., 2018). This conceptualization has been widely adopted in empirical research to differentiate the functions and intended objectives of diverse digital psychological intervention tools (Linardon et al., 2019). Against this backdrop, closer attention is warranted for a core psychological process widely regarded as pivotal to intervention effectiveness in these tools: self-disclosure.

With the rapid growth of digital mental health tools and AI conversational agents, the role of self-disclosure (SD) in psychological interventions has been increasingly emphasized. Classic work has long indicated that individuals’ open expression of emotions, thoughts, and distress in a safe context has been conceptualized as an important mechanism for psychological healing and relational repair (Derlega and Chaikin, 1977; Jourard, 1971). Compared with research emphasizing functional utility or technology acceptance, self-disclosure more directly reflects whether users are willing to articulate emotions, thoughts, and personal concerns during chatbot interactions; it has been widely regarded as a central pathway through which psychological relief and affective restoration occur (Greene et al., 2006). More recent studies on digital mental health agents and AI conversational agents suggest that users’ willingness to engage in emotional and private self-disclosure during interaction has been identified as a key prerequisite for intervention effectiveness (Li et al., 2023; Balan et al., 2024). Longitudinal and empirical research on the development of human–chatbot relationships has shown that self-disclosure has been associated with psychological closeness and companionship and with sustained use intentions; it has also been shown to be shaped by self-compassion, sense of meaning, and factors such as culture and gender (Skjuve et al., 2021; Xie et al., 2025). In addition, recent work on digital “relational agents” and emotionally supportive chatbots has indicated that appropriate emotion-expression strategies and humanlike cues may reduce users’ psychological resistance, thereby facilitating self-disclosure and emotional sharing (Sha et al., 2025; Warren-Smith et al., 2025). Nevertheless, a considerable portion of the literature has continued to focus on technology-acceptance determinants such as perceived usefulness, perceived ease of use, and trust (Davis, 1989; Venkatesh et al., 2003; Müller et al., 2023). Comparatively less attention has been devoted to the underlying psychological mechanisms and antecedents of self-disclosure in mental health contexts, thereby constraining, to some extent, a deeper understanding of why and how chatbot-based mental health interventions work.

Although research on chatbot-based mental health interventions has advanced, several gaps remain. First, most studies have been confined to a single type of system or interaction form, and systematic comparisons across text-, voice-, and multimodal configurations have remained scarce (Norouzi et al., 2022; Balan et al., 2024). Consequently, the literature has been limited in explaining how configuration-specific differences—such as interface cues, anthropomorphism, and interaction pacing—collectively shape users’ psychological and behavioral responses (Li et al., 2023). Second, technology-acceptance factors (e.g., perceived usefulness, ease of use, and trust) have been emphasized, whereas self-disclosure—arguably more central in mental health contexts—has received comparatively less attention (Davis, 1989; Venkatesh et al., 2003). In particular, research providing a systematic account of why users are willing to confide in chatbots from a disclosure-mechanism perspective has remained limited (Li et al., 2023). Third, prior psychological and behavioral studies have predominantly relied on linear modeling approaches, which often fail to capture potential nonlinear relationships between interaction configurations and psychological mechanisms; moreover, the necessary role of certain psychological variables in self-disclosure has seldom been examined (e.g., whether emotional arousal is a prerequisite for self-disclosure). Although Necessary Condition Analysis (NCA) has been increasingly applied in behavioral research (González et al., 2008; Dul, 2016), it has rarely been applied in chatbot-based mental health settings. Therefore, to unpack the complex pathway through which interaction configurations influence users’ self-disclosure via psychological mechanisms, a research framework is needed that can simultaneously reveal linear, nonlinear, and necessary relationships.

The Stimulus–Organism–Response (S–O–R) model is a classic theoretical framework in which behavioral responses are posited to be influenced by external stimuli through individuals’ internal psychological processing (Zhao et al., 2020). In the present study, an overall real-world configuration—rather than an isolated modality—was used as the unit of analysis. Accordingly, three representative configurations of mental health AI chatbots were operationalized as distinct external stimuli: a text-based configuration (Wysa), a voice-based configuration (ChatGPT-5), and a multimodal/digital-human configuration (Replika). Emotional arousal, affective engagement, and psychological acceptance were specified as key organism-level mechanisms, whereas self-disclosure was specified as the behavioral response. Although chatbot design features have been suggested to shape user experience and affective states, empirical evidence remains limited regarding how these specific configurations systematically influence willingness to self-disclose through mechanisms such as emotional responses and acceptance. Based on this observation, a multi-stage mixed-method approach—structural equation modeling (SEM), artificial neural networks (ANN), and Necessary Condition Analysis (NCA)—was implemented. By integrating sufficiency and necessity logics, differences across the three configurations in emotional responses and self-disclosure mechanisms were examined more systematically.

Building on the above background and gaps, the present study was designed to systematically examine how three representative configurations of mental health AI chatbots—Wysa as a text-based configuration, ChatGPT-5 as a voice-based configuration, and Replika as a multimodal configuration—are associated with university students’ willingness to self-disclose through psychological mechanisms. Specifically, three research questions were formulated:

*RQ1*: To what extent are emotional arousal, affective engagement, and psychological acceptance shaped by the interaction approach and interface features of the three configurations?

*RQ2*: Are emotional arousal, affective engagement, and psychological acceptance positioned as mediators between configuration differences and self-disclosure, and how do the pathways differ across mechanisms?

*RQ3*: Can a multi-method modeling strategy (SEM, ANN, and NCA) be used to identify nonlinear relationships and necessary conditions between configurations and psychological mechanisms, thereby addressing the limitations of a single linear model?

By addressing these questions, contributions are provided at the theoretical, methodological, and practical levels. Theoretically, the S–O–R model was extended to mental health AI chatbots by focusing on three real-world system–modality configurations. The mechanisms through which different configurations are associated with self-disclosure via emotional arousal, affective engagement, and psychological acceptance were clarified, and a more ecologically grounded, configuration-level perspective was introduced to interaction research. Methodologically, SEM, ANN, and NCA were integrated to move beyond single linear analyses, providing a comprehensive approach for examining linear associations, nonlinear patterns, and necessary conditions; this framework is expected to provide stronger explanatory and predictive power for complex psychological–behavioral mechanisms. Practically, actionable implications are derived for the configuration and interaction design of mental health AI chatbots: by optimizing interaction approaches and interface design to enhance affective engagement and psychological acceptance, users’ willingness to self-disclose may be strengthened, thereby improving the real-world effectiveness of mental health interventions. It should be emphasized, however, that because interaction modality and platform-specific features were operationalized as bundled configurations in the experimental design, the conclusions should be interpreted as describing differences among real-world configurations rather than as definitive evidence regarding the causal effects of interaction modality *per se*.

## 2 Literature

### 2.1 Stimuli–Organism–Response (S–O–R) model

The Stimulus–Organism–Response (S–O–R) model is often traced to Woodworth and Schlosberg’s (1954) extension of the classic Stimulus–Response (S–R) perspective and has been widely used to explain how behavioral outcomes are shaped by external environmental cues through individuals’ internal psychological processing (Pavlov, 2010; Donovan and Rossiter, 2002; Pandita et al., 2021). The model emphasizes that behavioral responses are not determined directly by external stimuli; rather, they are generated via mediation and moderation by internal psychological processes, including cognition, emotion, and attitudes (Bagozzi, 1986). Structurally, the Stimulus (S) refers to external cues and design inputs embedded in an interaction context, such as interface cues, social cues, or interaction modalities (Eroglu et al., 2003). The Organism (O) denotes the individual’s internal processing of these stimuli, including perceptual, affective, and cognitive mechanisms (Pandita et al., 2021). The Response (R) represents behavioral or attitudinal outcomes that follow from these internal processes, including choices, satisfaction, continuance intention, or self-disclosure (Buxbaum, 2016; Fu et al., 2021).

The S–O–R model has been extensively applied in retail, e-commerce, social media, and service-experience research to examine how users’ emotional and behavioral responses are elicited by contextual cues (Chang et al., 2011). More recently, the model has been extended to healthcare and digital mental health intervention settings and has been increasingly adopted as a theoretical framework for explaining emotional responses, trust formation, and health-related behaviors (Suess and Mody, 2018). For example, Yang et al. (2021) showed that anxiety responses can be influenced by external stimuli through internal cognitive processing in health-anxiety research. Wang et al. (2024) found that avoidance behavior can be shaped by stimuli such as risk perception and information overload via cognitive dissonance and affective experiences in studies of health-information avoidance. Yuan et al. (2024) applied the S–O–R framework to postpartum depression interventions and reported that emotional states and coping strategies can be improved through problem-solving–oriented nursing grounded in this framework.

In the context of the present study, a clear pathway is provided by the S–O–R model for explaining how mental health AI chatbots may influence users’ self-disclosure. It should be emphasized that, using an applied and ecologically oriented approach, three representative interaction configurations of mental health chatbots—implemented as integrated “system + interaction mode” configurations—were operationalized as the external Stimulus (S). On this basis, the ways in which self-disclosure (R) may be influenced by these configurations through users’ internal psychological mechanisms (O) were examined. Accordingly, the conclusions are best interpreted as accounting for the combined differences among real-world configurations rather than as definitive evidence regarding the causal effects of any single modality in isolation.

#### 2.1.1 Applications of AI chatbots in mental health (stimuli)

AI-driven mental health chatbots are typically defined as conversational agents built on natural language processing and machine learning, with core functions that include delivering emotional relief, psychological support, or cognitive-behavioral interventions through dialogue-based interaction (Provoost et al., 2017; Abd-Alrazaq et al., 2019). In recent years, such tools have been increasingly deployed in contexts involving depression, anxiety, and stress management. To some extent, shortages in mental health service resources may be mitigated and accessibility and immediacy of supportive experiences may be improved through their use (Inkster et al., 2018; Abd-Alrazaq et al., 2020). Representative systems have included Wysa, Woebot, and Replika (Inkster et al., 2018; Laestadius et al., 2024; Yeh et al., 2025).

From the standpoint of real-world deployment, current mental health chatbots can be broadly characterized in terms of three typical interaction configurations. First, text-centered configurations have been widely adopted for companionship and early-stage intervention because they are relatively low cost and highly scalable (Park et al., 2021; Li et al., 2023). Second, voice-centered configurations are enabled by speech recognition and synthesis, through which more natural spoken communication may be supported and advantages in emotional expression and the transmission of social cues may be realized (Zhu et al., 2022). Third, multimodal configurations typically combine text, voice, and visual presentation (e.g., anthropomorphic avatars or digital humans) and are often posited to enhance immersion, social presence, and trust formation (Casu et al., 2024). At the same time, distinct limitations may be introduced by each configuration. For example, nonverbal cues may be absent in text-based interaction (Sharma et al., 2023); voice-based interaction may be constrained by recognition accuracy and environmental noise (Lee et al., 2020); and multimodal interaction may elicit the “uncanny valley” effect and heighten privacy concerns (Lima et al., 2021; Belen Saglam et al., 2021). Collectively, these differences indicate that further investigation is warranted in mental health contexts to determine how interaction configurations may shape users’ psychological responses and self-disclosure mechanisms.

From an S–O–R perspective, the interaction configurations described above can be treated as external stimuli (S). Through differences in interface cues, social cues, and feedback styles, internal psychological processing (O) may be elicited, and behavioral responses (R), including self-disclosure, may subsequently be shaped.

#### 2.1.2 Emotional arousal, affective engagement, and psychological acceptance (organism)

Within the S–O–R model, the Organism component typically refers to psychological and affective processes that are elicited by external stimuli and that shape behavioral responses through cognitive, emotional, and attitudinal pathways (Buxbaum, 2016). In the context of mental health AI chatbots, users’ self-disclosure often unfolds as a progressive sequence—from emotional triggering, to the development of an engaged interaction experience, and ultimately to the establishment of sufficient safety and trust to support open disclosure. On this basis, emotional arousal (EA), affective engagement (AE), and psychological acceptance (PA) were specified as the three core Organism-level variables. These constructs correspond to three complementary dimensions—(i) intensity of emotional activation, (ii) quality of engagement during interaction, and (iii) safety-based acceptance and trust—and were used to represent the key psychological mechanism chain through which interaction configurations may influence self-disclosure.

##### 2.1.2.1 Emotional arousal

Within the S–O–R model, emotional arousal has been conceptualized as a key psychological mechanism linking external stimuli to individual responses. Schachter and Singer (1962) proposed that emotion is jointly determined by physiological arousal and cognitive labeling, whereas Lazarus’s (1991) cognitive appraisal theory emphasized that emotional type and intensity are shaped by individuals’ evaluations of situational meaning. Related work has suggested that arousal and behavioral performance may exhibit nonlinear associations, as illustrated by the Yerkes–Dodson law (Yerkes and Dodson, 1908), consistent with the perspective of bounded rationality (Simon, 1997). Empirical evidence further indicates that motivation and psychological states can be shaped by arousal (Vieira, 2013; Yang et al., 2021; Yuan et al., 2024). Drawing on these perspectives, EA was defined as the intensity of emotional activation and level of alertness experienced during interaction with a mental health chatbot, with emphasis placed on an immediate activation state (rather than a hedonic evaluation of the interaction experience). Accordingly, it was posited that EA may be initially shaped by interface cues and feedback styles across configurations and may subsequently influence downstream psychological processes.

##### 2.1.2 2 Affective engagement

Affective engagement is commonly conceptualized as a sustained psychological response that is elicited by external stimuli. It reflects interest, pleasure, and immersion during interaction and may influence behavioral intentions and engagement depth (Fritsch, 2009). Norman’s (2004) affective design framework suggests that user experience is jointly shaped by visceral-level affect, behavioral-level usability and fluency, and reflective-level value endorsement and meaning construction. Empirical studies have further shown that anthropomorphic cues, pleasurable experiences, and feedback mechanisms may enhance user engagement and involvement (Grawemeyer et al., 2017; Ashfaq et al., 2020; Lee and Lee, 2023). In this study, AE was defined as interest, enjoyment, and immersion experienced during interaction with a mental health chatbot, emphasizing the process-oriented quality of engagement. Importantly, EA and AE are conceptually distinct: EA reflects the intensity of emotional activation and is typically short-lived and activation-oriented, whereas AE reflects whether interaction is engaging and supports sustained participation, and is formed gradually over continued interaction. On this basis, EA was treated as one potential foundation for AE, such that emotional activation (EA) may be shaped first by an interaction configuration and sustained affective involvement (AE) may be shaped subsequently.

##### 2.1.2.3 Psychological acceptance

In psychological research and cognitive-behavioral therapy, psychological acceptance has been regarded as an important coping mechanism for stress and distress, and it may function as a key mediator of psychological improvement during interventions (Herbert et al., 2009). In user experience research, acceptance has also been closely linked to instrumental and hedonic gratification, trust, and continued use (Hassenzahl, 2010; Hassenzahl and Tractinsky, 2006). Cross-context evidence suggests that when acceptance is higher and psychological resistance is lower, support-seeking and self-expression are more likely (Wang et al., 2025). Accordingly, PA was defined as the sense of trust, safety, and being understood formed toward the chatbot and its responses during interaction, reflecting the psychological safety basis for open and honest disclosure. Relative to EA and AE, PA was positioned as a more proximal psychological condition preceding self-disclosure: private and sensitive psychological content is more likely to be disclosed when acceptance and trust are perceived.

In sum, EA, AE, and PA characterize, respectively, emotional activation elicited by interaction stimuli, sustained engagement experience, and the safety-based foundation of acceptance. These constructs are interlinked yet functionally differentiated, jointly constituting the key psychological mechanism chain linking interaction configuration to self-disclosure. This conceptualization also provides the theoretical basis for subsequent multi-method analyses (SEM, ANN, and NCA) in which linear relationships, nonlinear patterns, and necessary conditions are examined.

##### 2.1.2.4 Self-disclosure (response)

Within the Stimulus–Organism–Response (S–O–R) framework, self-disclosure (SD) is conceptualized as a core behavioral response that emerges from the combined influence of external stimuli and internal psychological mechanisms. Psychological research has long identified self-disclosure not only as an essential means of forming interpersonal relationships but also as a central process through which individuals alleviate stress, seek support, and cultivate trust in social interactions (Jourard, 1971; Derlega and Chaikin, 1977). In digital mental health interventions, self-disclosure is particularly critical because users’ willingness to express emotions and experiences openly directly determines the intervention’s effectiveness and depth (Greene et al., 2006).

Previous studies have shown that interaction design, emotional mechanisms, and social context significantly affect the degree of self-disclosure. De Choudhury and De (2014), in an empirical analysis of Reddit, found that anonymity substantially reduced users’ perceived social risk, thereby encouraging more open sharing of psychological distress and facilitating emotional support. Yu et al. (2015) further revealed the emotional dimension of self-disclosure, showing that emotions not only directly drive disclosure behavior but also indirectly increase disclosure intention by amplifying perceived benefits. Lee et al. (2020) experimentally demonstrated that chatbots can function as intermediaries between users and mental health professionals, with variations in conversational style promoting deeper levels of self-disclosure. Similarly, Zhang (2017) reported that college students experiencing stress were more likely to disclose personal experiences on Facebook, and such disclosures served to buffer stress and enhance psychological wellbeing. In Eastern cultural contexts, self-disclosure is further shaped by factors such as stigma and mindfulness. Wang et al. (2025), drawing on the S–O–R framework and the mindfulness coping model, found that information exposure and trait mindfulness positively influenced Chinese adolescents’ willingness to engage in online counseling, whereas public and self-stigma significantly reduced this willingness. These findings suggest that cultural values and social stigma constitute significant barriers to self-disclosure.

Taken together, self-disclosure functions as both a determinant of intervention effectiveness and a central construct in digital interaction research. Anonymity, emotional experience, conversational style, and cultural influences collectively shape users’ willingness to disclose, ultimately determining the degree to which trust and psychological acceptance are established within mental health interventions.

### 2.2 Hypothesis development

#### 2.2.1 Emotional arousal

Different interaction modalities are known to elicit users’ emotional arousal through distinct sensory cues. In the context of text-based interaction, prior studies have indicated that this mode is widely adopted owing to its anonymity and controllability, which help reduce social pressure and communication anxiety while enhancing users’ willingness for self-expression. Joinson’s (2001) experimental findings revealed that anonymity in text-based environments not only alleviates concerns about social evaluation but also facilitates self-disclosure and emotional expression. Similarly, Brandtzaeg and Følstad (2017) observed that users tended to exhibit more explicit emotional reactions when interacting with text-based chatbots.

By contrast, voice-based interaction relies on acoustic features—such as tone, pauses, and prosody—that provide a more vivid channel for emotional expression. Nass and Brave (2005) experimentally demonstrated that, compared with text-only interfaces, voice interfaces significantly heighten emotional arousal. Likewise, Lopatovska et al. (2019), in a field study of Amazon Alexa, found that users frequently experienced heightened emotional responses during voice-based interactions, influenced by factors such as vocal tone, response speed, and speech style. These human-like vocal cues not only enhance the sense of immersion during interaction but also foster a perceived social presence comparable to that of interpersonal communication. Extending this to avatar-based or multimodal interaction, these formats integrate embodied visual appearances and observable facial expressions alongside speech, thereby offering richer social cues through the combination of visual and auditory stimuli. Existing research consistently indicates that multimodal interaction significantly enhances emotional arousal through the integration of visual, auditory, and nonverbal signals. For instance, de Melo et al. (2019) demonstrated that the emotional expressiveness of virtual agents’ facial cues directly affected users’ emotional states and decision-making behaviors. Similarly, Zhang J. et al. (2024), in a large-scale interaction analysis, reported that compared with text-only dialogues, multimodal interactions significantly improved user retention and conversation duration, effectively eliciting emotional arousal through greater immersion and enhanced information comprehension. Based on the above discussion, the following hypotheses are proposed:

*H1a*: Text-based interaction exerts a significant positive effect on users’ emotional arousal (EA).

*H1b*: Voice-based interaction exerts a significant positive effect on users’ emotional arousal (EA).

*H1c*: Multimodal interaction exerts a significant positive effect on users’ emotional arousal (EA).

#### 2.2.2 Affective Engagement

In text-based interaction, linguistic cues function not only as vehicles for conveying information but also as stimuli that elicit users’ emotional engagement. Zhang L. et al. (2024) found in an experimental study that embedding emojis or images within text exchanges significantly enhanced users’ perceptions of human-likeness and social interactivity, primarily through the mediating role of social presence. Similarly, Soderlund et al. (2021) reported that even in purely text-based communication, incorporating expressions of happiness significantly improved users’ positive evaluations of the interaction. These findings suggest that text-based interaction is far from neutral or impersonal; instead, it stimulates users’ interest and engagement through emotionally expressive language, thereby fostering affective involvement.

In voice-based interaction, the influence of nonverbal features becomes even more salient. Zhu et al. (2022), through experimental manipulation of a voice assistant’s prosody and intonation, found that emotional vocal outputs enhanced users’ perception of emotional expressiveness and increased their engagement, sense of anthropomorphism, and overall likability. This finding suggests that voice-based interaction effectively activates users’ affective engagement by cultivating a heightened sense of social presence. Additional large-scale evidence was provided by Maduku et al. (2024), who analyzed 125,600 Google Assistant reviews and found that positive emotions and cognitive absorption were key psychological drivers of deep user engagement. These results indicate that the value of voice-based interaction extends beyond recognition accuracy; its true contribution lies in eliciting users’ emotional and psychological engagement. In multimodal interaction, the integration of linguistic and visual channels provides a more immersive experience. Wang et al. (2025) developed the ArtTheraCat system, demonstrating that integrating supportive dialogue with AI-generated art images significantly improved users’ emotional states and facilitated emotional externalization and self-insight through nonverbal channels. Similarly, Yu et al. (2024) found that the embodied chatbot Haru, which combined vocal and facial expressions, significantly enhanced users’ sense of immersion and naturalness; participants reported notably higher affective engagement compared with interactions involving neutral-type robots.

Taken together, different interaction modalities display a progressive hierarchy of emotional expressiveness—from text to voice to multimodal formats—each eliciting affective engagement to varying degrees through distinct sensory cues. This progression underscores the central role of affective engagement in interactive processes and establishes a theoretical foundation for designing AI chatbots for mental health applications.

*H2a*: Text-based interaction exerts a significant positive effect on users’ affective engagement (AE).

*H2b*: Voice-based interaction exerts a significant positive effect on users’ affective engagement (AE).

*H2c*: Multimodal interaction exerts a significant positive effect on users’ affective engagement (AE).

#### 2.2.3 Psychological acceptance

In text-based interaction settings, Ashfaq et al. (2020) found that information and service quality significantly enhanced user satisfaction, whereas perceived enjoyment, usefulness, and ease of use served as strong predictors of continued usage intention. These findings suggest that text-based interaction fosters psychological acceptance by enhancing user satisfaction and trust. Cheng et al. (2022) further demonstrated that users’ perceptions of chatbot empathy and friendliness significantly increased trust and reduced resistance during interaction, thereby providing empirical support for H3a. Within the context of voice-based interaction, psychological acceptance is largely driven by users’ perceived social presence. Zhan et al. (2024), based on an empirical analysis of 300 users, reported that practicality, credibility, and privacy protection significantly strengthened user trust, which in turn functioned as a key mechanism driving adoption. Similarly, Sezgin et al. (2020) reviewed prior research and found that voice assistants exhibited high feasibility and user satisfaction in health intervention settings, thus providing further evidence for H3b. In multimodal interaction, the integration of textual, vocal, and visual cues has been shown to enhance users’ sense of immersion and empathetic responsiveness. Chu et al. (2025) developed the SMES framework, which significantly increased users’ trust and acceptance. Likewise, Holla et al. (2025) introduced the Mental Health Companion system, demonstrating that multimodal interaction effectively establishes a trustworthy and supportive interaction environment, thereby providing support for H3c.

*H3a*: Text-based interaction exerts a significant positive effect on users’ psychological acceptance (PA).

*H3b*: Voice-based interaction exerts a significant positive effect on users’ psychological acceptance (PA).

*H3c*: Multimodal interaction exerts a significant positive effect on users’ psychological acceptance (PA).

#### 2.2.4 Self-disclosure

In virtual reality research, Wang et al. (2025) found that intelligent virtual agents (IVAs) incorporating different emotional tones during self-disclosure significantly affected the depth of relationship formation with children. High-valence emotions fostered closer interpersonal bonds, whereas low-arousal negative emotions more effectively elicited empathy and increased the likelihood of self-disclosure. This finding underscores the pivotal role of emotional arousal in the self-disclosure process, suggesting that the degree of emotional activation experienced during interaction directly shapes users’ willingness to disclose. Extending this perspective to conversational AI, Saffarizadeh et al. (2024) demonstrated the impact of reciprocal self-disclosure: when chatbots proactively revealed personal information, users not only became more willing to engage in responsive self-disclosure but also developed stronger trust, driven by heightened anthropomorphic perception. This mechanism indicates that emotional arousal and affective engagement often function synergistically in eliciting self-disclosure.

A comparable phenomenon has also been observed in online mental health communities. Zhang et al. (2025) introduced the MentalImager system, which generates emotion-related images to help users externalize feelings more naturally, thereby reducing barriers to self-disclosure. User studies revealed that such emotionally charged visual stimuli not only enhanced disclosure satisfaction but also fostered supporters’ empathy and willingness to provide help. Taken together, evidence from virtual agents, text- and voice-based chatbots, and generative multimodal systems consistently demonstrates that emotional arousal facilitates self-disclosure across diverse interaction contexts.

*H4*: Users’ emotional arousal (EA) exerts a significant positive effect on self-disclosure (SD).

In digital healthcare interactions, affective engagement often serves as a crucial bridge between user participation and self-disclosure. Drawing on an internet-based cognitive behavioral therapy (iCBT) framework, Goh et al. (2023) conducted an experiment comparing two dialogue script versions—with and without self-disclosure components. The results indicated that dialogues incorporating self-disclosure significantly increased users’ engagement, perceived system quality, and intention to continue use, whereas non-disclosure dialogues were perceived as more task-oriented, thereby diminishing the overall interaction experience. This contrast suggests that when users become more emotionally engaged, they are also more inclined to share personal experiences and emotions, thereby enhancing self-disclosure. This relationship has also been validated in large-scale online community studies. Lee et al. (2023) analyzed 2,399 posts and nearly 30,000 comments from mental health support communities during the COVID-19 pandemic and found that higher levels of self-disclosure were associated with greater informational support and more active engagement indicators, such as submission scores, comment counts, and comment length. Although the role of self-disclosure varied across informational and emotional support contexts, the overall trend indicated that deeper disclosure corresponded to more positive interaction experiences, indirectly confirming the importance of affective engagement in facilitating disclosure behaviors.

At the design level, Park et al. (2023) developed the Emoware system, which visualized predicted audience emotions to reduce uncertainty and social anxiety during text-based communication. Experimental results revealed that users of this system were not only more willing to share personal information but also exhibited greater focus and immersion during writing tasks. In other words, affective engagement not only enhances interaction quality but also serves as an intrinsic psychological driver of self-disclosure.

*H5*: Users’ affective engagement (AE) exerts a significant positive effect on self-disclosure (SD).

In empirical studies on adolescent mental health services, Bradford and Rickwood (2015) tested the electronic psychosocial tool myAssessment and found that it was not only widely accepted but also significantly increased adolescents’ self-disclosure regarding sensitive topics such as substance use, sexual behavior, and self-harm—with reporting rates 2.78–10.38 times higher than those of the control group. These results indicate that when users experience trust and comfort with a system, psychological acceptance effectively reduces disclosure anxiety and promotes more authentic expression.

Similar patterns have been observed in studies involving children’s interactions with smart speakers. Lee et al. (2023) found through field experiments that both robot-initiated and user-mediated disclosures stimulated children’s self-disclosure, and that multi-robot environments could either amplify or attenuate this effect. These findings suggest that psychological acceptance functions as a key mechanism enabling users to share personal information and emotions in robot-mediated interactions. Evidence from social media studies provides further support. Forest and Wood (2012), examining individuals with low self-esteem, found that Facebook’s text-based environment was perceived as a safe space for self-disclosure due to reduced perceived social risk, thereby encouraging more open expression of personal information. Although disclosures from low self-esteem individuals tended to be more negative and received less favorable feedback, the findings nonetheless emphasized that psychological acceptance and perceived safety are crucial prerequisites for self-disclosure.

Across these domains—clinical interventions, child–robot interaction, and social media use—psychological acceptance consistently emerges as a fundamental mechanism that enhances users’ willingness to disclose by fostering trust, comfort, and perceived safety.

*H6*: Users’ psychological acceptance (PA) exerts a significant positive effect on self-disclosure (SD).

### 2.3 Conceptual framework

**FIGURE 1:**
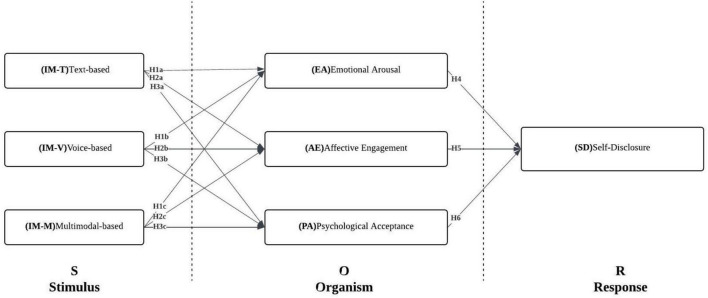
Hypothesized path model.

Building on the preceding discussion, the conceptual framework of this study is presented in Figure 1. It is hypothesized that the three interaction modalities of AI chatbots—text-based, voice-based, and avatar-based (H1–H3)—have significant impacts on users’ emotional arousal, affective engagement, and psychological acceptance. Furthermore, as posited in Hypotheses H4–H7, these psychological perceptions and interaction experiences are expected to influence users’ behavioral responses, particularly by enhancing their willingness to self-disclose.

## 3 Research methodology

### 3.1 Research process

**FIGURE 2:**
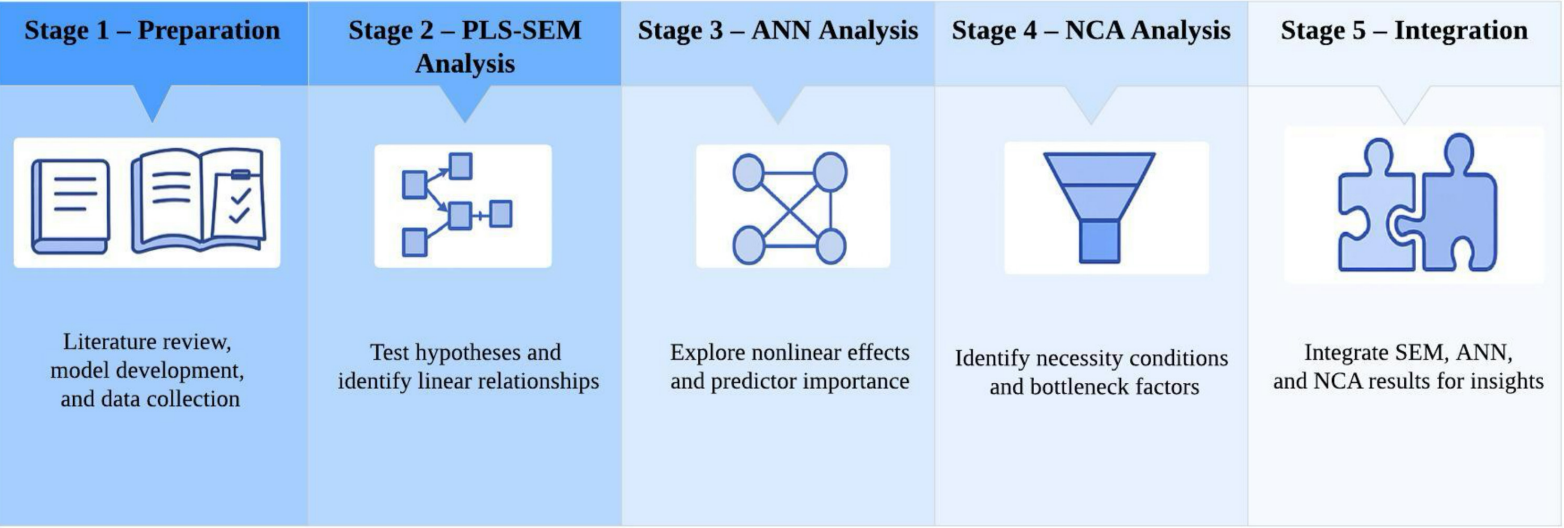
Research process chart.

This study was carried out in five sequential stages, as depicted in Figure 2. In the first stage, a systematic literature review was conducted to identify the key factors influencing users’ interactions with AI chatbots. Drawing on these findings, a conceptual model was developed, followed by the design of the survey instrument, data collection, and descriptive statistical analysis of valid responses. In the second stage, the Partial Least Squares Structural Equation Modeling (PLS-SEM) approach was employed to test the hypothesized relationships within the conceptual model, with particular emphasis on identifying the linear paths among variables. Moreover, the “sufficiency logic” of PLS-SEM was applied to determine which factors, when present, were sufficient to guarantee the occurrence of the outcome. In the third stage, latent variable scores derived from the PLS-SEM were used as inputs for an Artificial Neural Network (ANN) to further examine nonlinear relationships among variables and rank the relative importance of predictors, enabling a comparative evaluation of PLS-SEM and ANN results. In the fourth stage, a Necessary Condition Analysis (NCA) was conducted to identify, from the perspective of “necessity logic,” the essential preconditions for the occurrence of the outcome—specifically, those factors whose absence would prevent it. The results of PLS-SEM and NCA were subsequently integrated to refine the conceptual model, thereby differentiating between necessary and sufficient key factors. Finally, in the fifth stage, findings from the SEM, ANN, and NCA analyses were synthesized to derive theoretical and practical implications, providing a comprehensive explanation of how different interaction modes influence users’ self-disclosure through emotional arousal, affective engagement, and psychological acceptance.

#### 3.1.1 Experimental procedure

**FIGURE 3:**
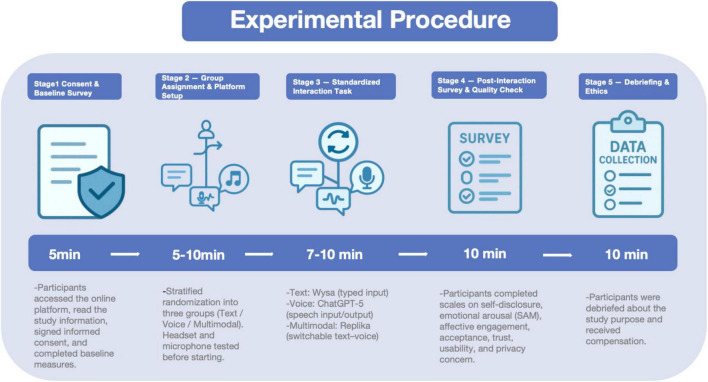
Experimental procedure.

During the interaction phase Figure 3, participants in the text-based condition interacted with the chatbot *Wysa* via typed messages; those in the voice-based condition communicated with *ChatGPT-5* using microphone-based speech input and audio output; and those in the multimodal condition used *Replika*, which allowed free switching between text and voice with real-time dual-channel feedback. All three systems were standardized with identical prompts and conversational scripts to ensure content consistency and control for medium-related variations.

### 3.2 Data collection

#### 3.2.1 Pre-research analysis

To ensure sufficient statistical power, an a priori power analysis was performed using G*Power 3.1 (Faul et al., 2009) prior to data collection. Given a significance level of α = 0.05, an effect size of *f* = 0.40 (Cohen, 2013), and a statistical power of (1–β) = 0.95, the minimum required sample size for a three-group comparison (ANOVA) was calculated as *N* = 102. The final dataset comprised 352 valid responses, exceeding the minimum threshold and thereby enhancing the robustness and reliability of subsequent statistical analyses.

The questionnaire used in this study consisted of three main sections. The first section presented the informed consent procedure and screened participants according to their prior experience with managing or using mental health applications. Individuals who declined consent or failed to meet this criterion were excluded from further participation. The second section collected demographic information, including gender, age, educational level, and recent stress level. The third section included measurement items corresponding to the seven constructs defined in the research model.

Table 1 provides a summary of the conceptual definitions, item counts, measurement statements, and corresponding source references for each construct. All measurement items were adapted and refined from established scales and underwent content validity evaluation by psychological counselors and academic advisors at a university counseling center to ensure conceptual clarity, relevance, and contextual appropriateness. Responses were recorded using a five-point Likert scale (1 = strongly disagree; 5 = strongly agree).

**TABLE 1:** Measurement variables and their references.

Dimension	Code and content	Sources
Interaction mode—text-based (IM-T)	IM-T1: This interaction mode makes me feel natural and clear. IM-T2: This interaction mode makes it easier for me to express myself. IM-T3: This interaction mode helps me better understand the dialogue content. IM-T4: This interaction mode gives me a stronger sense of immersion. IM-T5: This interaction mode enhances my communication experience with the chatbot.	(Gefen et al., 2003) (Brandtzaeg and Følstad, 2017) (Joinson, 2001) (Wiederhold and Riva, 2019) (Araujo, 2018)
Interaction mode—voice-based (IM-V)	IM-V1: This interaction mode makes me feel natural and clear. IM-V2: This interaction mode makes it easier for me to express myself. IM-V3: This interaction mode helps me better understand the dialogue content. IM-V4: This interaction mode gives me a stronger sense of immersion. IM-V5: This interaction mode enhances my communication experience with the chatbot.	(Lopatovska and Oropeza, 2018) (Cowie et al., 2001) (Eyben et al., 2015) (Wiederhold and Riva, 2019) (Araujo, 2018)
Interaction mode—multimodal (IM-M)	IM-M1: This interaction mode makes me feel natural and clear. IM-M2: This interaction mode makes it easier for me to express myself. IM-M3: This interaction mode helps me better understand the dialogue content. IM-M4: This interaction mode gives me a stronger sense of immersion. IM-M5: This interaction mode enhances my communication experience with the chatbot.	(McDonnell et al., 2012) (Garau et al., 2003) (de Melo et al., 2014) (Wiederhold and Riva, 2019) (Araujo, 2018)
Emotional arousal (EA)	EA1: I felt emotionally aroused while interacting with the chatbot. EA2: Using the chatbot made me feel more focused and alert. EA3: The conversation with the chatbot evoked strong emotional reactions.	(Bradley and Lang, 1994) (Chen et al., 2025) (Wang and Peng, 2023))
Affective engagement (AE)	AE1: The interaction with the chatbot made me feel immersed in the experience. AE2: I felt strong interest and engagement while interacting with the chatbot. AE3: Using the chatbot was enjoyable and appealing to me.	(O’Brien and Toms, 2010) (Yeast, 2025) (Becker et al., 2025))
Psychological acceptance (PA)	PA1: I am willing to trust the advice or responses provided by the chatbot. PA2: I can comfortably accept the process of interacting with the chatbot. PA3: Communication with the chatbot makes me feel sfe and understood.	(Venkatesh et al., 2003) (Koulouri et al., 2022) (Li et al., 2024) (Inkster et al., 2018)
Self-disclosure (SD)	SD1: I am willing to express my personal feelings in conversations with the chatbot. SD2: I am willing to share my stress or concerns with the chatbot. SD3: I tend to reveal some private information during interactions with the chatbot.	(Lee and Lee, 2023) (Myers and Johnson, 2004) (Wheeless et al., 1986)

### 3.3 Interaction conditions and stimulus materials

**FIGURE 4:**
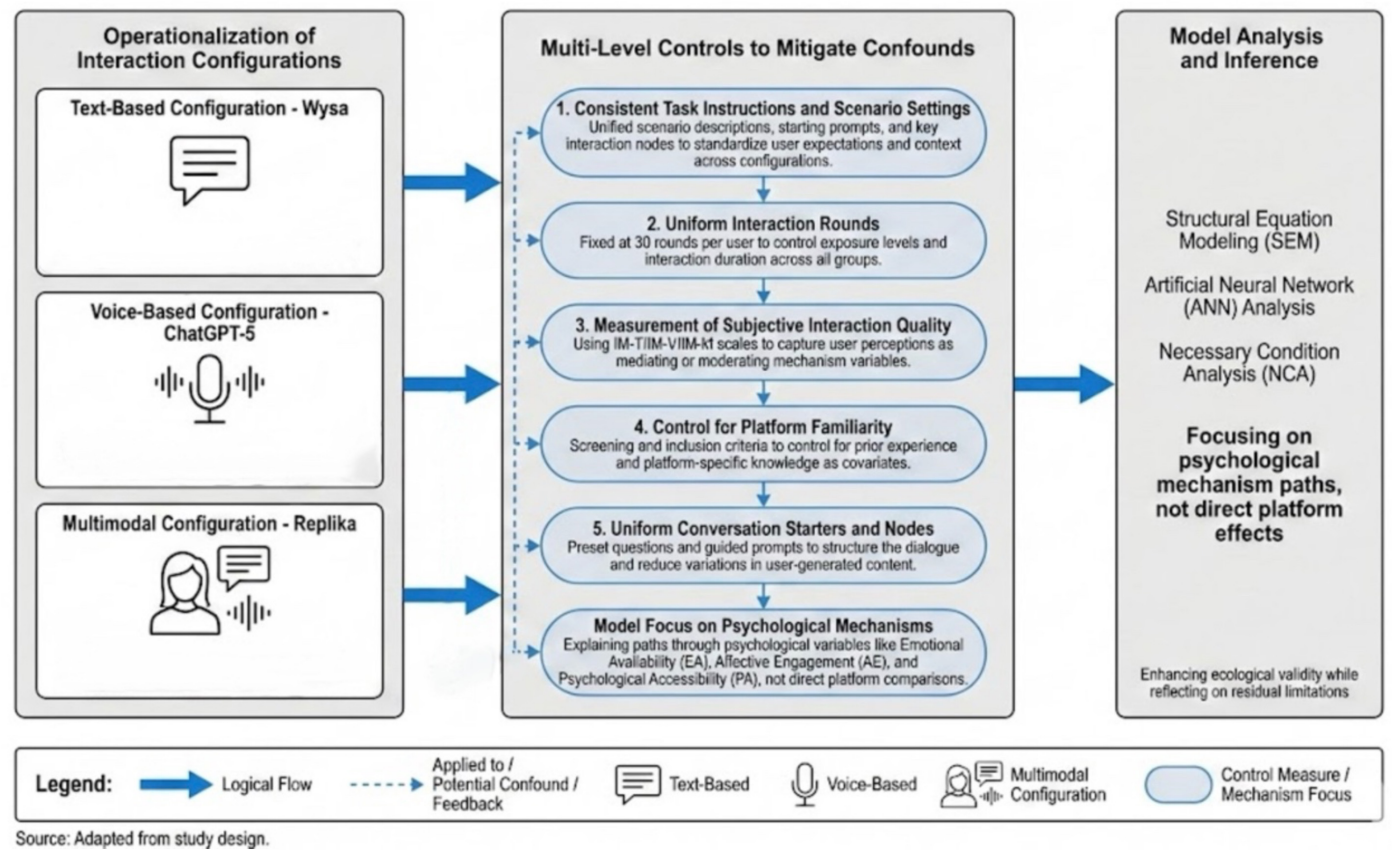
Experimental tools and experimental screening process diagram

In the present study, the “interaction configuration” was operationalized as three representative mental health AI chatbot configurations: a text-based configuration, a voice-based configuration, and a multimodal configuration. Specifically, the text condition was implemented using Wysa for text-only dialogue; the voice condition was implemented using the voice interface of ChatGPT-5; and the multimodal condition was implemented using Replika, which features an anthropomorphic avatar and a combined text-and-voice interface. As shown in Figure 4, these three systems were selected to represent three common real-world configurations in mental health chatbot applications, rather than for toggling interface modalities within the same underlying model. It should be noted that although ecological validity was enhanced by this design, interaction modality was unavoidably bundled with system-level characteristics (e.g., algorithmic capability, degree of anthropomorphism, and interface design style). To minimize confounding risks attributable to differences across systems, multiple control procedures were implemented:

First, highly consistent task instructions and contextual framing were maintained across the three conditions. Before the formal interaction, all participants were asked to read the same scenario description: “Please recall a specific event from the recent past that caused substantial stress or emotional fluctuation, and discuss this event with the chatbot.” A standardized opening prompt was provided across groups; for example, participants were asked to briefly describe the event background, their emotional experience at the time, and the aspects that remained distressing. During subsequent interactions, several scripted checkpoints were incorporated (e.g., prompting participants to elaborate on emotional experiences or to express previously unarticulated thoughts), and the same prompting logic was applied across the three configurations. This scripted framework and dialogue structure were intended to keep conversation topics, information triggers, and emotional foci as consistent as possible across configurations, thereby reducing confounding introduced by differences in system-generated content.

Second, the number of interaction turns was standardized to control exposure levels. To ensure comparability across configurations, the number of turns per participant was standardized to 30 in the assigned chatbot (i.e., approximately 30 response units). When system replies were relatively brief, participants were reminded to continue elaborating on the same event; when replies were lengthy, the conversation was guided to the next scripted checkpoint. By standardizing both the number of turns and the approximate interaction duration, interaction intensity and experiential richness were kept at a comparable level across the three groups.

Third, participants’ subjective perceptions of interaction quality were measured and incorporated into subsequent analyses as key explanatory variables. After completing the chatbot interaction, interaction-quality scales corresponding to the three configurations (IM-T, IM-V, and IM-M) were completed by all participants. These instruments were used to assess perceived interaction quality in terms of naturalness, clarity, ease of expression, ease of understanding, and immersion. These indicators were used to compare overall differences in interaction experience across configurations and were also incorporated into subsequent models as key psychological mechanism variables. This approach enabled effects on emotional arousal and self-disclosure to be interpreted through perceived experience, rather than treating platform identity as a proxy for superiority or inferiority.

Fourth, differences in platform familiarity were controlled. Because prior experience with some platforms may have been present, a pre-interaction screening item assessing “platform use experience” was administered to reduce bias attributable to familiarity differences. Participants were asked to report prior use of mental health chatbots and whether systems such as Wysa, ChatGPT, or Replika had been heard of or used. Based on these reports, “platform familiarity” was included as a control variable in the structural equation model and in robustness checks. The results indicated that inclusion of platform familiarity did not change the significance or direction of key paths, suggesting that the findings were not driven solely by familiarity and strengthening the credibility of the inferences.

Fifth, the conversation opening and key interaction checkpoints were standardized to attenuate variation in system-generated content. A uniform conversation-opening structure was specified across configurations: each system initiated the interaction with a “welcome + brief statement of purpose + invitation to share a recent stress-related event” prompt. In subsequent dialogue, deeper disclosure was encouraged using predetermined questions (e.g., “Which aspect of this event affected you the most?” “What was the strongest emotion you felt at that time?” “Are there thoughts you usually would not say out loud?”). The semantic content of these core questions was kept consistent across the three conditions, with only minor wording adjustments made to accommodate text, voice, and multimodal interfaces. In this way, while system-specific generative capabilities were retained, consistency in conversational structure and focus was maximized, thereby reducing confounding effects arising from variation in content-generation style.

Finally, psychological mechanisms—rather than direct “platform identity” effects—were emphasized in the modeling strategy and analytic interpretation. It should be emphasized that platform identity (i.e., dummy-coded indicators for Wysa/ChatGPT/Replika) was not used as the primary predictor of self-disclosure in the structural equation model or in subsequent ANN and NCA analyses. Instead, pathways from configuration to self-disclosure were modeled through psychological mechanism variables, including emotional arousal (EA), affective engagement (AE), psychological acceptance (PA), and perceived interaction quality. Platform identity was used only as a condition variable in between-group comparisons and robustness analyses, rather than as a basis for evaluating overall system superiority. This specification helped keep the analytical focus on psychological mechanisms and reduced the risk that overall between-system differences would be conflated with causal effects of interaction modality *per se*.

Overall, a real-world perspective was adopted by treating three typical “system + interaction mode” configurations of mental health AI chatbots as stimulus conditions. Through task scripting, standardized interaction turns, subjective experience measurement, familiarity controls, and mechanism-oriented modeling, confounding influences attributable to system-specific features were reduced as far as feasible. The corresponding design choices and residual limitations are described and discussed in the Discussion and Limitations sections.

### 3.4 Respondent profile

A convenience-sampling strategy combined with online recruitment was used to obtain the study sample. Recruitment announcements were posted on a campus forum at a designated university, and respondents were directed to the questionnaire only after informed consent had been read and provided. To ensure that responses were grounded in actual usage experience, a screening criterion requiring prior use of mental health–related applications or services was implemented; respondents who did not meet this criterion were prevented from proceeding. To enhance data quality, attention-check items were embedded in the questionnaire by using explicit response instructions (e.g., “To ensure response accuracy, please select the specified option”) to identify inattentive responding. After data collection, invalid responses were removed according to predefined criteria, including missing values on key items, anomalous completion time, clearly patterned responding, and failed attention checks. The final valid sample comprised *N* = 352 respondents and was used for subsequent analyses. The study protocol was approved by the local ethics committee.

As shown in Table 2, the sample included 69.89% male respondents (*n* = 246) and 30.11% female respondents (*n* = 106). The age distribution was concentrated in the 19–21 age group (40.06%, *n* = 141), followed by 25–27 (30.11%, *n* = 106) and ≥ 28 years (19.89%, *n* = 70), with 22–24 accounting for 9.94% (*n* = 35). All respondents were undergraduates; third-year students constituted the largest proportion (40.06%, *n* = 141), followed by first-year students (20.17%, *n* = 71), whereas second-year and fourth-year students each accounted for 19.89% (*n* = 70). With respect to academic disciplines, science and engineering majors accounted for 49.72% (*n* = 175), humanities majors for 19.89% (*n* = 70), arts and design majors for 9.94% (*n* = 35), and other majors for 20.45% (*n* = 72).

**TABLE 2:** Demographic characteristics of respondents.

Attribute	Code	Numbers	Percentage (%)
Gender	Male	246	69.89
	Female	106	30.11
Age	≤ 18 years	0	0.00
	19–21 years	141	40.06
	22–24 years	35	9.94
	25–27 years	106	30.11
	≥ 28 years	70	19.89
Academic status	Undergraduate, year 1	71	20.17
	Undergraduate, year 2	70	19.89
	Undergraduate, year 3	141	40.06
	Undergraduate, year 4	70	19.89
	Graduate (master’s/other)	0	0.00
Field of study	Science and engineering	175	49.72
	Humanities	70	19.89
	Arts and design	35	9.94
	Medical sciences	0	0.00
	Other	72	20.45
Use of mental health apps	Never	70	19.89
	Occasionally	141	40.06
	Frequently	141	40.06
Perceived stress in the past 30 days	None	70	19.89
	Mild	71	20.17
	Moderate	0	0.00
	High	140	39.77
	Extreme	71	20.17

Regarding mental health application use, 80.12% of respondents reported having used such applications (never used: 19.89%, *n* = 70), and the proportions reporting “occasional use” and “frequent use” were identical (40.06% each; *n* = 141). With respect to perceived psychological stress over the past 30 days, relatively high proportions reported high stress (39.77%, *n* = 140) and extremely high stress (20.17%, *n* = 71). In contrast, no stress and mild stress were reported by 19.89% (*n* = 70) and 20.17% (*n* = 71), respectively, whereas moderate stress was not reported in this sample (0%).

It should be noted that the sample exhibited a gender imbalance (69.89% male). Because online convenience sampling was used, participants were primarily recruited via a specific university campus forum, and prior experience with mental health applications was required as an eligibility criterion, participation and response rates may have differed across gender groups, thereby producing a male-skewed sample. Accordingly, this gender distribution is more likely to reflect structural features of the recruitment source and sampling frame than the gender composition of the broader university-student population. The implications of this gender imbalance for generalizability are further discussed in the Limitations and Future Research Directions section.

## 4 Results overview and analytical strategy

To address the research questions systematically and to overcome the limitations of relying on a single analytic technique for modeling complex psychological mechanisms, a multi-method framework comprising PLS-SEM, ANN, and NCA was adopted in the present study. This framework was not implemented as a set of parallel analyses; rather, it was organized sequentially to follow a “theory testing–nonlinearity augmentation–necessity identification” logic, so that explanatory and predictive strengths could be leveraged complementarily.

First, PLS-SEM was used to test the structural model and hypotheses derived from the S–O–R framework. Because multiple latent constructs and their interrelationships can be handled simultaneously, linear effects and mediation mechanisms linking interaction configurations (Stimulus) to self-disclosure (Response) via emotional arousal, affective engagement, and psychological acceptance (Organism) can be evaluated, thereby directly addressing RQ1 and RQ2. However, because PLS-SEM is grounded in assumptions of linearity and compensatory relationships, potential nonlinear structures in complex interaction settings may be underestimated.

To address this limitation, artificial neural network (ANN) analysis was further introduced. Because ANN is not constrained by linearity assumptions, potential nonlinear and non-compensatory relationships among variables can be captured, and predictive performance can be evaluated. In the present study, ANN models were built using paths that were significant in the PLS-SEM results as inputs. The relative importance of different interaction configurations and psychological mechanisms under nonlinear conditions was then examined, thereby providing complementary evidence for identifying the nonlinear component of RQ3.

Finally, Necessary Condition Analysis (NCA) was applied to identify minimum threshold conditions that must be satisfied for outcomes such as self-disclosure to occur, using a logic distinct from correlation- and regression-based approaches. NCA focuses on whether an outcome is impossible in the absence of a given condition, and thus can reveal necessity structures that cannot be identified by SEM or ANN. In this way, the necessary-condition component of RQ3 was addressed. By integrating NCA with the sufficiency-based evidence provided by PLS-SEM, it was possible to distinguish which psychological mechanisms function as facilitators and which operate as constraining thresholds.

In sum, a multi-layered and complementary analytic framework was established through a sequential strategy—PLS-SEM (explanation) → ANN (prediction) → NCA (threshold identification)—thereby providing more comprehensive empirical evidence regarding how different interaction configurations influence self-disclosure through psychological mechanisms.

### 4.1 PLS-SEM results

The variance inflation factors (VIFs) of all latent variables within the inner model were examined to assess potential multicollinearity. According to Kock (2015), when all VIF values derived from the full collinearity test are equal to or less than 3.3, the model is considered free of common method bias (CMB). In this study, the VIF values for all latent variables ranged between 1.157 and 1.506 (see Table 3), suggesting that common method bias was negligible in the proposed model.

**TABLE 3:** Reflective scale accuracy analyses.

Variables	Code	Factor loading	Cronbach’s alpha (α)	CR	AVE
AE	AE-1	0.858	0.880	0.917	0.735
	AE-2	0.844			
	AE-3	0.863			
	AE-4	0.865			
EA	EA-1	0.902	0.877	0.924	0.803
	EA-2	0.897			
	EA-3	0.889			
IMM	IMM-1	0.884	0.920	0.940	0.758
	IMM-2	0.870			
	IMM-3	0.853			
	IMM-4	0.868			
	IMM-5	0.879			
IMT	IMT-1	0.869	0.909	0.932	0.734
	IMT-2	0.852			
	IMT-3	0.798			
	IMT-4	0.900			
	IMT-5	0.860			
IMV	IMV-1	0.885	0.915	0.936	0.746
	IMV-2	0.857			
	IMV-3	0.847			
	IMV-4	0.885			
	IMV-5	0.842			
PA	PA-1	0.867	0.858	0.904	0.701
	PA-2	0.824			
	PA-3	0.821			
	PA-4	0.835			
SD	SD-1	0.876	0.858	0.904	0.702
	SD-2	0.803			
	SD-3	0.820			
	SD-4	0.849			

#### 4.1.1 Assessment of measurement model

According to Sarstedt et al. (2021), standardized factor loadings in the outer measurement model should be greater than 0.71, which indicates that the corresponding squared multiple correlations (SMCs) exceed 0.50. As suggested by Hair et al. (2021), the minimum acceptable value for composite reliability (CR) and internal consistency reliability (Cronbach’s alpha) is 0.70. Convergent validity was assessed using the average variance extracted (AVE), which should exceed 0.50 (Fornell and Larcker, 1981). The results for indicator reliability, composite reliability (CR), internal consistency reliability (Cronbach’s alpha), and convergent validity (AVE) are presented in Table 3, indicating that all measurement items satisfied the recommended thresholds.

Discriminant validity is defined as the degree to which a latent construct is empirically distinct from other constructs within the structural model. It was evaluated using the heterotrait–monotrait ratio (HTMT) approach proposed by Henseler et al. (2014), which recommends that HTMT values remain below 0.85. Furthermore, discriminant validity was also examined using the Fornell–Larcker criterion (Fornell and Larcker, 1981), which specifies that the square root of the average variance extracted (AVE) for each construct should exceed its correlations with all other constructs in the model. The results of the discriminant validity analysis are reported in Table 4, demonstrating that all constructs in this study satisfied the recommended thresholds.

**TABLE 4:** Inter-structural correlation matrix, HTMT statistic.

	AE	EA	IMM	IMT	IMV	PA	SD
AE	0.857	0.346	0.514	0.454	0.472	0.400	0.431
EA	0.394	0.896	0.503	0.466	0.500	0.254	0.470
IMM	0.571	0.559	0.871	0.526	0.454	0.457	0.459
IMT	0.507	0.519	0.575	0.857	0.456	0.431	0.399
IMV	0.524	0.555	0.492	0.496	0.863	0.511	0.380
PA	0.461	0.289	0.514	0.485	0.574	0.837	0.430
SD	0.495	0.540	0.513	0.447	0.423	0.497	0.838

#### 4.1.2 Assessment of structural model

In the inner-model assessment, a bias-corrected and accelerated (BCa) bootstrapping procedure with 5,000 resamples was applied to estimate path coefficients and their statistical significance. In addition, consistent with Hair et al. (2019), multicollinearity was examined prior to evaluating the structural relationships to reduce the risk of biased coefficient estimates. Kock proposed that, in PLS-SEM, inner-model VIF values below 3.3 indicate the absence of problematic collinearity. In the present model, the VIF values among all latent constructs ranged from 1.157 to 1.506 (see Table 5), indicating that no meaningful multicollinearity was present in the inner model.

**TABLE 5:** Model path coefficients and their significance, VIF.

Hypotheses	(β)	Standard deviation	*T*	*P*-values	Support	VIF	
AE -> SD	0.212	0.051	4.122	0.000	Yes	1.288	0.054
EA -> SD	0.330	0.053	6.260	0.000	Yes	1.157	0.147
IMM -> AE	0.305	0.061	5.012	0.000	Yes	1.501	0.097
IMM -> EA	0.271	0.064	4.204	0.000	Yes	1.501	0.078
IMM -> PA	0.219	0.050	4.364	0.000	Yes	1.501	0.048
IMT -> AE	0.179	0.058	3.073	0.002	Yes	1.506	0.033
IMT -> EA	0.192	0.068	2.805	0.005	Yes	1.506	0.039
IMT -> PA	0.162	0.051	3.177	0.001	Yes	1.506	0.027
IMV -> AE	0.252	0.059	4.304	0.000	Yes	1.373	0.072
IMV -> EA	0.289	0.064	4.526	0.000	Yes	1.373	0.097
IMV -> PA	0.338	0.058	5.798	0.000	Yes	1.373	0.127
PA -> SD	0.262	0.050	5.246	0.000	Yes	1.212	0.088

**FIGURE 5:**
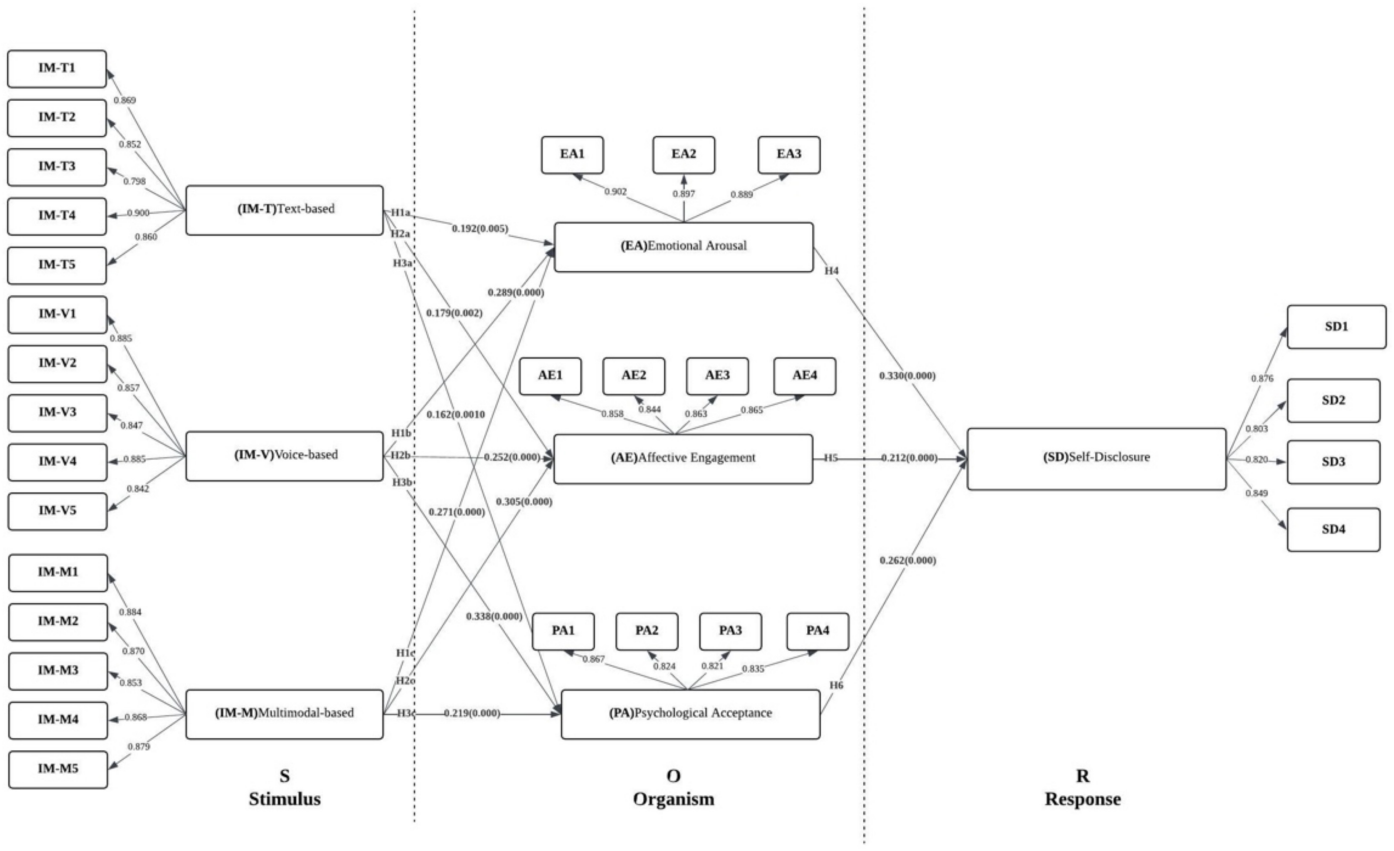
Structural model evaluation results.

Figure 5 presents the structural path results estimated within the S–O–R framework. Overall, acceptable model fit was indicated, and all hypothesized paths were supported with statistically significant coefficients. At the Stimulus (S) level, the three interaction configurations—text (IMT), voice (IMM), and multimodal (IMV)—were each associated with significant positive effects on Organism (O)-level mechanisms, including emotional arousal (EA), affective engagement (AE), and psychological acceptance (PA). Among these effects, the multimodal configuration (IMV) showed the strongest association with psychological acceptance (β = 0.338, *t* = 5.798, *p*< 0.001). Significant effects on affective engagement and emotional arousal were also observed for the voice configuration (IMM) (β = 0.305, *t* = 5.012; β = 0.271, *t* = 4.204; both *p* < 0.001). Positive effects on all three psychological mechanisms were likewise observed for the text configuration (IMT) (β range = 0.162–0.192; all significant). Collectively, these findings indicate that interaction configurations, as external stimuli, are capable of eliciting users’ emotional and psychological responses.

At the Organism (O) level, affective engagement (AE), emotional arousal (EA), and psychological acceptance (PA) were each positively associated with self-disclosure (SD) at the Response (R) level. The strongest effect on SD was observed for EA (β = 0.330, *t* = 6.260, *p* < 0.001), followed by PA (β = 0.262, *t* = 5.246, *p* < 0.001) and AE (β = 0.212, *t* = 4.122, *p* < 0.001). These results suggest that, during interaction with mental health chatbots, emotional activation and perceived acceptance may represent key psychological mechanisms through which users’ willingness to self-disclose is facilitated. Overall, the results supported the robustness of the proposed model and underscored the importance of AE, EA, and PA in explaining SD, whereas IMM, IMT, and IMV served as significant predictors of AE, EA, and PA. In addition, to assess overall approximate model fit, the standardized root mean square residual (SRMR) was computed. SRMR captures the average discrepancy between the observed correlation matrix and the model-implied correlation matrix and is widely used as an overall fit index in PLS-SEM (Garson, 2016). Prior research has suggested that SRMR values below 0.08 indicate acceptable model fit (Henseler et al., 2014). In the present study, the estimated SRMR was 0.060, indicating acceptable overall model fit.

Furthermore, all path-related variance inflation factor (VIF) values ranged from 1.157 to 1.506, which are well below the conventional thresholds (5 or 10), indicating the absence of multicollinearity. Overall, these results confirm the robustness of the structural model and highlight the pivotal roles of AE, EA, and PA in explaining SD, whereas IMT, IMM, and IMV act as significant predictors of AE, EA, and PA.

To further assess the model’s explanatory power and predictive performance, the coefficients of determination (R^2^), predictive relevance (Q^2^), effect size (*f*^2^), and goodness-of-fit (GoF) indices were computed for all endogenous latent constructs (see Supplementary Figure 1). The results indicated that AE (*R*^2^ = 0.357), EA (*R*^2^ = 0.370), PA (*R*^2^ = 0.343), and SD (*R*^2^ = 0.359) exhibited moderate explanatory power, consistent with the thresholds proposed by Chin (1998). Furthermore, all Q^2^ values exceeded zero, confirming the model’s satisfactory predictive relevance, with EA (Q^2^ = 0.353) demonstrating the strongest predictive capability. The effect sizes (*f*^2^) for the structural paths ranged primarily from 0.027 to 0.147, suggesting that most relationships exhibited small to medium magnitudes. The overall GoF value of 0.514 further confirmed a high level of model quality (Hair et al., 2013) (see Table 6).

**TABLE 6:** The values of R2 Q2.

Constructs	R2	Remarks	Q2	Predictive relevance	Remarks
AE	0.357	Moderate	0.341	Q2>0	Moderate
EA	0.370	Moderate	0.353	Q2>0	Strong
PA	0.343	Moderate	0.330	Q2>0	Moderate
SD	0.359	Moderate	0.254	Q2>0	Moderate

Furthermore, as presented in Table 7, the mediating effects were tested using the bias-corrected and accelerated (BCa) bootstrapping procedure with 5,000 resamples. The results revealed that the impacts of all three interaction modalities—text-based (IMT), voice-based (IMM), and multimodal (IMV)—on self-disclosure (SD) were mediated by emotional arousal (EA), affective engagement (AE), and psychological acceptance (PA), with all indirect paths reaching statistical significance (*p* < 0.01). Among these, multimodal interaction demonstrated the strongest total indirect effect (β = 0.237, *t* = 8.318, *p* < 0.001). These findings further support the theoretical proposition of the S–O–R framework, which posits that psychological mechanisms mediate the relationship between interaction modality and self-disclosure.

**TABLE 7:** Analysis of specific and total mediating effects.

Specific indirect effects	Original sample (O)	Standard deviation	T statistics	*P*-values
IMM -> PA -> SD	0.057	0.019	3.082	0.002
IMT -> PA -> SD	0.042	0.016	2.649	0.008
IMV -> PA -> SD	0.088	0.021	4.114	0.000
IMM -> EA -> SD	0.089	0.027	3.305	0.001
IMM -> AE -> SD	0.065	0.022	2.964	0.003
IMT -> EA -> SD	0.063	0.026	2.478	0.013
IMT -> AE -> SD	0.038	0.016	2.435	0.015
IMV -> EA -> SD	0.096	0.023	4.129	0.000
IMV -> AE -> SD	0.053	0.019	2.809	0.005
**Total indirect effects**	**Original sample (O)**	**Standard deviation**	**T statistics**	***P*-values**
IMM -> SD	0.211	0.033	6.465	0.000
IMT -> SD	0.144	0.032	4.434	0.000
IMV -> SD	0.237	0.029	8.318	0.000

### 4.2 ANN results

Because Structural Equation Modeling (SEM) assumes linear and compensatory relationships among latent variables, this assumption has increasingly been challenged in recent years (Lee et al., 2020). In practice, users’ psychological and behavioral processes often display complex nonlinear dynamics that may not be fully captured within the linear constraints of SEM. To address this limitation and improve the model’s predictive performance, Artificial Neural Network (ANN) analysis was employed to identify potential nonlinear and non-compensatory relationships among variables.

Following established research designs (Peng et al., 2025), the SEM results served as input data for training Multilayer Perceptron (MLP) networks in SPSS 27.0. Specifically, four independent ANN models were developed and tested (see Supplementary Figure 1) to predict the nonlinear effects of emotional arousal, affective engagement, psychological acceptance, and self-disclosure. Each model employed a Sigmoid activation function and the backpropagation algorithm to minimize prediction errors and optimize overall model performance.

#### 4.2.1 Validation of ANN

After identifying significant paths from the PLS-SEM analysis, a feedforward backpropagation Multilayer Perceptron (MLP) network was employed to examine and predict potential nonlinear and non-compensatory relationships (Taneja and Arora, 2019). Following the procedure outlined by Liébana-Cabanillas et al. (2017), latent variable scores generated from the PLS-SEM were utilized as input and output nodes in the ANN models. Four separate models were trained in SPSS 27.0: M1 (IMT, IMM, IMV → EA), M2 (IMT, IMM, IMV → AE), M3 (IMT, IMM, IMV → PA), and M4 (EA, AE, PA → SD). Both the hidden and output layers adopted Sigmoid activation functions, and the model training process was conducted using 10-fold cross-validation. Predictive performance was assessed using the root mean square error (RMSE) metric (Hyndman and Koehler, 2006). As presented in Table 8, all four models yielded consistently low RMSE values across both training and testing datasets: M1 (0.147 ± 0.003 / 0.140 ± 0.013), M2 (0.119 ± 0.004 / 0.108 ± 0.013), M3 (0.123 ± 0.002 / 0.109 ± 0.015), and M4 (0.124 ± 0.002 / 0.129 ± 0.011). These findings suggest that the models demonstrated a high level of predictive accuracy.

**TABLE 8:** RMSE values for ANN models 1–4.

Neural network	Model 1 (IMT, IMM, IMV → EA)		Model 2 (IMT, IMM, IMV → AE)		Model 3 (IMT, IMM, IMV → PA)		Model 4 (EA, AE, PA → SD)	
	Training	Testing	Training	Testing	Training	Testing	Training	Testing
ANN1	0.1451	0.1375	0.114	0.116	0.124	0.096	0.122	0.139
ANN2	0.1450	0.1553	0.111	0.119	0.123	0.105	0.124	0.116
ANN3	0.1488	0.1296	0.124	0.113	0.125	0.110	0.124	0.138
ANN4	0.1466	0.1378	0.118	0.088	0.124	0.094	0.124	0.146
ANN5	0.1557	0.1577	0.120	0.131	0.118	0.147	0.122	0.137
ANN6	0.1493	0.1176	0.122	0.102	0.123	0.112	0.129	0.135
ANN7	0.1450	0.1312	0.120	0.108	0.122	0.101	0.124	0.120
ANN8	0.1473	0.1521	0.124	0.097	0.126	0.119	0.125	0.122
ANN9	0.1449	0.1383	0.120	0.113	0.124	0.101	0.125	0.124
ANN10	0.1448	0.1468	0.116	0.094	0.123	0.106	0.125	0.112
**Mean**	**0.147**	**0.140**	**0.119**	**0.108**	**0.123**	**0.109**	**0.124**	**0.129**
**SD**	**0.003**	**0.013**	**0.004**	**0.013**	**0.002**	**0.015**	**0.002**	**0.011**

Mean is the average training and testing error across 10 independent neural network runs under the same model configuration, and SD indicates error variability, with smaller values reflecting greater model stability and robustness.

Following model construction, sensitivity analyses were performed for Models 1–4, as shown in Table 9. Sensitivity analysis is a key method for assessing the influence of input variables on model outputs (Nourani and Fard, 2012). In the context of Artificial Neural Networks (ANNs), sensitivity analysis serves as a valuable complement to PLS-SEM by capturing nonlinear relationships among variables, thereby offering additional empirical validation of SEM results (Chong, 2013).

**TABLE 9:** Sensitivity analysis results for ANN models 1–4.

Model	Output	Independent variables	Average RI	Normalized RI (%)	Ranking
Model 1	EA	IMT	0.286	75.4	3
		IMM	0.331	87.2	2
		IMV	0.383	100.0	1
Model 2	AE	IMT	0.228	53.1	3
		IMM	0.437	100.0	1
		IMV	0.335	77.6	2
Model 3	PA	IMT	0.212	47.0	3
		IMM	0.323	70.0	2
		IMV	0.465	100.0	1
Model 4	SD	AE	0.267	71.2	3
		EA	0.379	100.0	1
		PA	0.353	93.3	2

RI, Relative importance.

For Model 1 (EA prediction), multimodal interaction (IMV) showed the highest normalized importance (100.0%) for predicting emotional arousal, followed by voice-based interaction (IMM, 87.2%) and text-based interaction (IMT, 75.4%). For Model 2 (AE prediction), voice-based interaction (IMM) emerged as the most influential predictor (100.0%), followed by multimodal interaction (IMV, 77.6%) and text-based interaction (IMT, 53.1%). For Model 3 (PA prediction), multimodal interaction (IMV) was the most influential factor (100.0%), followed by voice-based interaction (IMM, 70.0%) and text-based interaction (IMT, 47.0%). Finally, for Model 4 (SD prediction), emotional arousal (EA) was identified as the most important predictor (100.0%), followed by psychological acceptance (PA, 93.3%) and affective engagement (AE, 71.2%).

These results underscore the distinct predictive contributions of each interaction modality and psychological mechanism, further confirming the robustness and validity of the integrated SEM–ANN analytical approach.

These results suggest that different interaction modalities exert differential effects on users’ psychological responses. Voice-based and multimodal interactions were found to be more influential than text-based interaction, whereas emotional arousal and psychological acceptance emerged as the most critical determinants of users’ self-disclosure behavior. These findings further support the S–O–R theoretical proposition that organism-level variables mediate the relationship between external stimuli and behavioral responses.

### 4.3 NCA results

To identify the necessary conditions required for outcome variables to reach relatively high levels, Necessary Condition Analysis (NCA) was conducted using the NCA package in R, which provides statistics such as effect size (d), accuracy, and permutation-test *p*-values (Dul et al., 2020). The permutation test was performed with 5,000 random permutations to construct a null distribution and evaluate the statistical significance of the effect size. To ensure consistency with the structural-model analysis, both the predictors and outcomes in the NCA were operationalized using latent variable scores estimated via PLS-SEM.

Ceiling lines were estimated using Ceiling Regression–Free Disposal Hull (CR-FDH), which is suitable for data that exhibit a quasi-continuous distribution after score transformation (Dul, 2016). Although the original indicators were measured on five-point Likert scales, the estimated latent variable scores can take intermediate values; therefore, CR-FDH was considered appropriate for ceiling-line estimation. The corresponding NCA scatterplots are reported in Supplementary Figure 2. The identification of necessary conditions followed a dual-criterion rule: a predictor was classified as a substantively meaningful necessary condition only when *d* ≥ 0.10 and the permutation test indicated *p* < 0.05. When the permutation test was significant but *d* < 0.10, the result was interpreted as statistically detectable but insufficiently constraining and was not treated as evidence of a necessary condition (Dul, 2016; Dul et al., 2020). Unlike PLS-SEM, which primarily characterizes average linear effects under a sufficiency logic, NCA is designed to identify the minimum threshold conditions that must be met for an outcome to occur at a given level, thereby complementing the evidence derived from PLS-SEM.

#### 4.3.1 Effect size and significance testing

Table 10 reports the effect size results obtained from the Necessary Condition Analysis (NCA), conducted using the ceiling regression–free disposal hull (CR-FDH) method. The analysis revealed distinct variations in necessary conditions across the examined target variables.

**TABLE 10:** NCA effect sizes (method: CR-FDH).

Target variable: SD	Effect size	Accuracy	Permutation *p*-value	Remarks
AE	0.027	99.715	0.687	No
EA	0.099	98.575	0.011	No
IMM	0.158	97.721	0.000	Yes
IMT	0.134	97.721	0.010	Yes
IMV	0.105	98.575	0.006	Yes
PA	0.170	97.721	0.000	Yes
**Target variable: EA**	**Effect size**	**Accuracy**	**Permutation *p*-value**	**Remarks**
IMM	0.040	100.000	0.452	No
IMT	0.049	100.000	0.047	No
IMV	0.000	100.000	0.691	No
**Target variable: AE**	**Effect size**	**Accuracy**	**Permutation *p*-value**	**Remarks**
IMM	0.183	96.866	0.000	Yes
IMT	0.199	95.726	0.018	Yes
IMV	0.156	97.151	0.012	Yes
**Target variable: PA**	**Effect size**	**Accuracy**	**Permutation *p*-value**	**Remarks**
IMM	0.080	99.145	0.007	No
IMT	0.088	98.860	0.006	No
IMV	0.071	99.715	0.000	No

First, when self-disclosure (SD) was designated as the outcome variable, voice-based interaction (IMM; *d* = 0.158, *p* = 0.000), text-based interaction (IMT; *d* = 0.134, *p* = 0.010), multimodal interaction (IMV; *d* = 0.105, *p* = 0.006), and psychological acceptance (PA; *d* = 0.170, *p* = 0.000) were identified as significant necessary conditions. This finding suggests that these factors must be present for individuals to achieve a certain level of SD, and the absence of any single factor would constrain its occurrence. In contrast, affective engagement (AE; *d* = 0.027, *p* = 0.687) and emotional arousal (EA; *d* = 0.099, *p* = 0.011) did not meet the necessity threshold, indicating that they do not act as constraining conditions for SD.

Second, when emotional arousal (EA) was treated as the dependent variable, none of the predictors—including IMM (*d* = 0.040, *p* = 0.452), IMT (*d* = 0.049, *p* = 0.047), and IMV (*d* = 0.000, *p* = 0.691)—satisfied the necessity criterion, suggesting that EA does not exhibit any significant necessary conditions. This implies that the development of EA may depend primarily on sufficient rather than necessary antecedents.

Third, for affective engagement (AE), the results indicated that IMM (*d* = 0.183, *p* = 0.000), IMT (*d* = 0.199, *p* = 0.018), and IMV (*d* = 0.156, *p* = 0.012) emerged as significant necessary conditions. This finding suggests that AE cannot reach a high level in the absence of these factors, underscoring their constraining influence on its development.

Finally, when psychological acceptance (PA) served as the dependent variable, although IMM (*d* = 0.080, *p* = 0.007), IMT (*d* = 0.088, *p* = 0.006), and IMV (*d* = 0.071, *p* = 0.000) achieved statistical significance, their effect sizes did not meet the necessity threshold. Therefore, PA cannot be regarded as possessing any strictly necessary conditions.

#### 4.3.2 Bottleneck analysis

Bottleneck analysis is used to determine the minimum threshold of predictor variables necessary to achieve a specific target level. Accordingly, bottleneck analyses were conducted for each dependent variable in the model to further quantify the necessity relationships (see Tables 11, 12). Both outcome and predictor variables were expressed as percentages.

**TABLE 11:** Bottleneck for SD (in percentage).

	AE	EA	IMM	IMT	IMV	PA
0.000%	–	–	0.000	0.000	0.000	0.000
10.000%	–	–	0.000	0.000	0.000	0.000
20.000%	–	–	0.000	0.000	0.000	0.000
30.000%	–	–	0.000	0.000	0.000	0.000
40.000%	–	–	0.000	0.000	0.000	0.000
50.000%	–	–	0.000	1.140	0.000	1.994
60.000%	–	–	0.285	5.698	0.000	5.128
70.000%	–	–	10.256	11.681	0.000	12.536
80.000%	–	–	26.781	16.524	14.530	26.781
90.000%	–	–	55.271	31.054	39.031	35.897
100.000%	–	–	78.632	44.444	77.778	63.248

**TABLE 12:** Bottleneck for AE (in percentage).

	IMM	IMT	IMV
0.000%	0.000	0.000	0.000
10.000%	0.000	0.000	0.000
20.000%	0.000	0.000	0.000
30.000%	0.000	0.000	0.000
40.000%	0.000	0.000	0.000
50.000%	0.000	1.140	0.000
60.000%	0.000	7.977	2.279
70.000%	10.256	20.798	13.960
80.000%	39.886	37.037	30.199
90.000%	77.208	64.672	52.707
100.000%	97.721	86.040	77.208

The results indicated that achieving a high level of self-disclosure (SD; ≥ 80%) required at least 26.781% of voice-based interaction (IMM), 16.524% of text-based interaction (IMT), 14.530% of multimodal interaction (IMV), and 26.781% of psychological acceptance (PA). These threshold values increased progressively with higher target levels. At the maximum SD level (100%), the minimum thresholds were 78.632% for IMM, 44.444% for IMT, 77.778% for IMV, and 63.248% for PA. No necessity thresholds were identified for affective engagement (AE) or emotional arousal (EA), suggesting that these factors do not impose strict constraints on SD. These findings reinforce the critical necessity of IMM, IMT, and PA in shaping self-disclosure.

Furthermore, no variables were identified as necessary conditions for either EA or PA within the model. To achieve a high level of AE ( ≥ 80%), at least 39.886% of IMM, 37.037% of IMT, and 30.199% of IMV were necessary. These threshold values also increased with the target level, reaching minimum thresholds of 97.721% for IMM, 86.040% for IMT, and 77.208% for IMV at the maximum AE level (100%).

By integrating Partial Least Squares Structural Equation Modeling (PLS-SEM) and Necessary Condition Analysis (NCA), this study identifies the distinction between factors that influence the dependent variables as sufficient conditions and those required for their occurrence as necessary conditions (see Table 13). Specifically, PLS-SEM captures the average effects of predictor variables on outcome variables, whereas NCA determines the minimum conditions necessary for an outcome to reach a specific level. The integration of these two analytical approaches offers a more comprehensive understanding of the underlying psychological mechanisms.

**TABLE 13:** Comparison of PLS-SEM and NCA results: sufficient and necessary conditions.

Dependent variable	Independent variable	PLS-SEM results	Effect size	Permutation *p*-value	Interpretation
SD	AE	Significant	0.027	0.687	Increases in AE do not necessarily lead to higher SD; the outcome does not require a minimum AE level.
	EA	Significant	0.099	0.011	Increases in EA do not necessarily result in higher SD; the outcome does not depend on a minimum EA threshold.
AE	PA	Significant	0.170	0.000	Higher PA levels promote SD; however, this effect depends on meeting the threshold conditions of IMM, IMT, and IMV.
	IMM	Significant	0.183	0.000	Higher IMM levels enhance AE but require IMT and IMV to reach their threshold levels.
	IMT	Significant	0.199	0.018	Higher IMT levels increase AE but only when IMM and IMV thresholds are met.
	IMV	Significant	0.156	0.012	Higher IMV levels improve AE but depend on IMM and IMT meeting their threshold conditions.
EA	IMM	Significant	0.040	0.452	IMM increases do not necessarily enhance EA; no minimum IMM level is required for the outcome.
	IMT	Significant	0.049	0.047	IMT increases do not necessarily elevate EA; no minimum IMT threshold is required.
	IMV	Significant	0.000	0.691	IMV increases do not necessarily enhance EA; the outcome does not require a minimum IMV level.
PA	IMM	Significant	0.080	0.007	IMM increases do not necessarily lead to higher PA; the outcome does not depend on a minimum IMM threshold.
	IMT	Significant	0.088	0.006	IMT increases do not necessarily lead to higher PA; no minimum IMT threshold is required.
	IMV	Significant	0.071	0.000	IMV increases do not necessarily lead to higher PA; the outcome does not require a minimum IMV level.

## 5 Discussion

This study systematically examined the mechanisms through which different interaction configurations of mental health AI chatbots were associated with users’ self-disclosure, and the proposed hypotheses were empirically tested. By integrating emotional arousal, affective engagement, and psychological acceptance into the theoretical model, the mediating pathways through which emotional and cognitive processes may facilitate self-disclosure were further elucidated. In addition, a hybrid analytical approach combining structural equation modeling (SEM), artificial neural networks (ANN), and necessary condition analysis (NCA) was employed to triangulate and compare the findings, thereby enabling a dual examination of model robustness and theoretical explanatory power. It should be emphasized that interaction conditions were operationalized as three representative real-world “system + interaction mode” configurations; accordingly, the results are best interpreted as reflecting the combined effects of these configurations rather than as strict causal tests of interface modality per se.

### 5.1 RQ1: To what extent do interaction approaches and interface features influence users’ emotional arousal, affective engagement, and psychological acceptance across the three representative configurations?

Significant differences in users’ emotional and psychological responses were observed across the three representative interaction configurations. These differences are unlikely to reflect isolated statistical artifacts and instead are more plausibly attributable to psychological mechanisms embedded within specific interaction experiences. In the text-based configuration represented by Wysa, which is characterized by relatively “de-socialized” interaction features, a low-risk and controllable communication environment may be provided, such that emotions can be expressed more authentically without identity disclosure. This pattern is consistent with Joinson’s (2001) “reduced evaluation anxiety” hypothesis and aligns with Brandtzaeg and Følstad’s (2017) conclusions regarding the role of text-based chat in fostering psychological safety. SEM results further indicated a significant path coefficient between the text-based configuration and emotional arousal (β = 0.192, *p* < 0.01), suggesting that even in the absence of acoustic or visual cues, emotional activation may be elicited through self-regulation and reflective expression in text-based interaction.

In contrast, more “embodied” social cues were introduced in the voice-based configuration represented by ChatGPT-5. Emotional contagion conveyed through intonation, pauses, and vocal dynamics may shift the interaction experience from information exchange toward affective communication. Prior studies (Nass and Brave, 2005; Zhu et al., 2022) have shown that voice interfaces can enhance emotional responses and social presence. A similar pattern was observed: the voice-based configuration was associated with the strongest effect on affective engagement (β = 0.305, *p* < 0.001) and was identified as the most important predictor (100%) in the ANN models. These findings suggest that, within the configuration comparisons examined here, sustained engagement may be strengthened through voice-based social cues, thereby increasing affective engagement.

Building on this pattern, more complex psychological effects were observed in the multimodal/digital-human configuration represented by Replika. Unlike single-channel information transmission, a sense of “human-like co-presence” may be constructed through the integration of visual and auditory cues in multimodal systems. When multi-source signals are received simultaneously, semantic information may be complemented by perceptual cues that enhance the experience of being responded to. Prior research by de Melo et al. (2014) and Yu et al. (2024) has demonstrated that embodied cues can increase social presence and trust. This trend was corroborated: the multimodal configuration was associated with the strongest effect on psychological acceptance (β = 0.338, *p* < 0.001) and achieved the highest importance score (100%) in the ANN models. Overall, across the three representative configurations, richer interaction cues were associated with higher levels of psychological acceptance, thereby providing a stronger psychological-safety foundation for subsequent self-disclosure.

### 5.2 RQ2: Do emotional arousal, affective engagement, and psychological acceptance mediate the relationship between configuration differences and self-disclosure, and how do their pathways differ?

Emotional and cognitive processes do not operate in isolation but instead function as psychological bridges through which a shift from passive perception to active expression may occur. The results indicate that emotional arousal (EA), affective engagement (AE), and psychological acceptance (PA) served as significant mediators between configuration differences and self-disclosure (SD). Specifically, EA exhibited the strongest effect on SD (β = 0.330, *p* < 0.001), indicating that higher levels of emotional activation during interaction were associated with greater disclosure tendency and intensity. PA showed the second strongest effect (β = 0.262, *p* < 0.001), underscoring trust and psychological safety as critical foundations for sustained and deepened disclosure. AE (β = 0.212, *p* < 0.001), by contrast, functioned as a process-oriented driver through which interest, enjoyment, and immersion may be sustained, thereby increasing the likelihood of continued expression.

Importantly, these mechanisms do not form a simple linear mediation chain but instead appear to operate synergistically at different levels. The rich cues afforded by the multimodal configuration were associated with higher emotional activation and acceptance, which helps explain its higher total indirect effect (β = 0.237, *p* < 0.001). This pattern is consistent with Saffarizadeh et al. (2024), who argue that stronger relational cues and anthropomorphic features can facilitate emotional responsiveness and trust. Similarly, in the context of mental health interventions, Goh et al. (2023) noted that when emotional expression is encouraged, self-disclosure tends to occur more naturally and deeply. Taken together, these findings suggest that in mental health chatbot contexts, self-disclosure is facilitated less by functional efficiency alone than by the capacity for emotional experience to be activated while psychological safety and a sense of being understood are concurrently established.

### 5.3 RQ3: Can multi-method models (SEM, ANN, NCA) reveal nonlinear relationships and necessary conditions, thereby complementing the limitations of a single linear model

In psychological and behavioral research, linear assumptions are often adopted as pragmatic simplifications of reality. However, such assumptions may be insufficient for capturing the complexity of human–AI interaction. To uncover nonlinear structures underlying average effects, ANN and NCA were jointly employed to address a critical question: when emotional and interaction mechanisms intertwine, which factors are substitutable, and which are indispensable?

Initial insights were provided by the ANN results. Across four neural network models, root mean square error (RMSE) values were all below 0.15, indicating stable and reliable nonlinear prediction. In these models, the multimodal configuration (IMV) was consistently identified as the most important input variable, regardless of whether emotional arousal (EA) or psychological acceptance (PA) was specified as the target outcome. This pattern suggests a “non-compensatory” structure for certain psychological outcomes: even when other factors perform well, predicted responses may be substantially constrained when key experiential cues are insufficient. This finding aligns with Chong’s (2013) notion of non-compensatory pathways and implies that, in complex interactive systems, user experience may be driven by a small number of dominant cues rather than by the average contribution of multiple factors.

Necessity-level regularities were further illuminated by NCA. For self-disclosure (SD), IMM, IMT, IMV, and PA were identified as necessary conditions (effect sizes *d* = 0.105–0.170, *p* < 0.01), whereas AE and EA did not constitute strict thresholds. In other words, EA is more plausibly interpreted as a facilitator that enhances disclosure tendency and depth rather than as a minimum condition required for disclosure to occur. Bottleneck analysis further clarified this mechanism: when SD reached an 80% level, voice and multimodal inputs were required to reach at least 26.781 and 14.530%, respectively, and PA was required to reach 26.781%. These findings suggest that high-intensity disclosure typically requires sufficient social presence and psychological safety; in the absence of these necessary conditions, favorable performance on other factors is unlikely to compensate for the deficit.

A particularly noteworthy pattern was observed when SEM and NCA results were compared. Although EA significantly predicted SD in SEM (β = 0.330, *p* < 0.001), it was not identified as a necessary condition in NCA. This apparent discrepancy reflects differences in causal logic rather than a substantive contradiction. SEM captures average marginal contributions and path significance at the sample level, reflecting a sufficiency logic in which higher EA corresponds to higher overall levels of SD. NCA, by contrast, examines necessity logic by assessing whether SD can occur when a given condition is absent. Thus, a significant positive association between EA and SD does not imply that SD cannot occur in the absence of EA. Mechanistically, this pattern suggests multiple substitutable pathways (equifinality) to self-disclosure: when EA is low, disclosure may still occur due to goal-oriented motives (e.g., seeking advice or organizing distress) or higher psychological acceptance and trust (PA). When EA is high, however, it may function as an “amplifier,” shifting disclosure from surface-level expression to deeper and more private content, thereby contributing to the significant path observed in SEM. Accordingly, EA is better conceptualized as a facilitator of disclosure probability and intensity, whereas PA is more closely aligned with the minimum psychological threshold for disclosure to occur. These findings illustrate how combining SEM and NCA allows both the factors that significantly enhance SD (sufficiency) and the minimum conditions for SD (necessity) to be identified, thereby offering a more comprehensive account of self-disclosure mechanisms in mental health human–AI interaction.

More broadly, the explanatory boundaries of the S–O–R framework in mental health human–AI interaction are called into question by these results. Psychological mechanisms may be influenced by external stimuli through sufficiency-based pathways while simultaneously being constrained by necessity thresholds. In other words, when key stimuli and psychological safety reach a threshold, behavioral responses become more likely; beyond that threshold, the depth and intensity of disclosure may be further shaped by emotional activation. This dual structure of “threshold plus facilitation” provides a more nuanced lens for understanding psychological complexity and offers a tractable methodological pathway for future research on affective human–AI interaction.

### 5.4 Integrative reflections and implications

By systematically integrating PLS-SEM, artificial neural networks (ANN), and Necessary Condition Analysis (NCA), understanding of self-disclosure mechanisms in mental health AI chatbot research was advanced at both the theoretical and methodological levels. Relative to prior work relying on a single statistical framework, this multi-method integration not only increases confidence in the robustness of the proposed relationships but also differentiates the roles of psychological mechanisms with respect to two core questions: whether self-disclosure occurs and the extent to which it unfolds.

The primary theoretical contribution is reflected in a functional differentiation of Organism-level mechanisms within the S–O–R model. In traditional S–O–R research, Organism constructs are often treated as parallel mediators, with emphasis placed on whether they significantly predict the Response. However, convergent evidence across methods indicates that the psychological mechanisms underlying self-disclosure do not operate at the same functional level. Specifically, emotional arousal (EA) was identified as the strongest mean-level predictor of self-disclosure (SD) in PLS-SEM and was ranked as highly important in the ANN models; however, it was not identified as a necessary condition in NCA. This pattern suggests that EA is best characterized as a facilitative mechanism, such that disclosure tends to become more probable and more intense as EA increases, while EA is not a prerequisite for disclosure to occur. By contrast, psychological acceptance (PA) not only significantly predicted SD in SEM but was also identified by NCA as a necessary condition for achieving higher levels of disclosure, with a clear bottleneck threshold. This pattern is consistent with a threshold (gatekeeping) mechanism, whereby high-intensity disclosure becomes difficult to attain in the absence of adequate acceptance and psychological safety. Affective engagement (AE), in turn, primarily reflected a process-oriented mechanism, with its contribution expressed more through sustaining interaction continuity and enhancing experiential quality than through imposing binding constraints on disclosure outcomes.

Taken together, these findings extend the explanatory scope of the S–O–R model in mental health human–AI interaction contexts by indicating that the Organism layer is not merely a set of interchangeable mediators. Rather, it may be better understood as a structured system composed of mechanisms with distinct functional roles. This perspective also helps explain why some variables may be statistically significant yet not constitute necessary (“critical”) conditions for the outcome.

By juxtaposing SEM and NCA, the theoretical distinction between “significant effects” and “critical conditions” was further clarified. SEM identifies mean-level effects in the overall sample—namely, whether a factor is statistically associated with higher levels of the outcome under a sufficiency logic. NCA, by contrast, identifies the minimum constraints that must be met for the outcome to reach a specified level under a necessity logic.

Accordingly, the pattern whereby EA was significant in SEM but not necessary in NCA does not imply a theoretical inconsistency. Instead, it highlights a dual logic of self-disclosure: disclosure may occur across a range of arousal levels, but without adequate psychological acceptance (PA), deeper and more sensitive disclosure is unlikely to be achieved. This distinction provides an important theoretical insight into why individuals may be willing to disclose in mental health contexts, yet may not necessarily disclose at a deeper level.

Methodologically, the distinctive benefits of integrating multiple analytic approaches in the study of complex psychological mechanisms were demonstrated. PLS-SEM enables systematic tests of theorized paths and mediation relationships; ANN captures potential nonlinear and non-compensatory relationships while strengthening predictive assessment; and NCA reveals structural constraints and minimum thresholds that cannot be detected through association-based methods alone. Together, these approaches form a coherent analytic chain—mean-level effects → predictive importance → necessary thresholds—in which each method addresses the inferential limitations of the others.

This integration also reduces the risk of method-specific interpretive bias. Reliance on SEM alone may overemphasize variables that are statistically significant but not structurally critical; reliance on prediction models alone may weaken theoretical interpretability; and reliance on necessity analysis alone may understate the cumulative influence of facilitative mechanisms. By combining all three approaches, a more comprehensive characterization of psychological mechanisms across multiple inferential layers was enabled.

More broadly, the methodological approach adopted in this study can serve as a reusable analytic template for research in digital mental health and human–AI interaction. When psychological processes involve emotion, trust, and self-disclosure—constructs that are often complex and potentially nonlinear—single linear models may fail to fully represent the underlying dynamics. By embedding ANN and NCA within a theory-driven SEM foundation, key psychological conditions and design constraints can be identified while theoretical interpretability is preserved.

This template is particularly suitable for evaluating the combined effects of different technological configurations, interaction strategies, or intervention mechanisms, allowing researchers to address not only whether an approach is effective, but also the conditions under which it is effective and the elements whose absence may lead to failure.

Overall, a more structural understanding of the Organism layer in the S–O–R model was advanced by differentiating the functional roles of psychological mechanisms in the formation of self-disclosure. Methodologically, the added value of integrating PLS-SEM, ANN, and NCA was demonstrated for revealing complex mechanisms through complementary inferential logics. Together, these contributions support progress along three dimensions—explanation, prediction, and constraint identification—thereby providing a stronger foundation for subsequent theoretical development and empirical research in mental health AI interaction.

## 6 Implications, limitations, and conclusions

### 6.1 Implications

The findings of this study not only advance the understanding of interaction mechanisms in mental health chatbots but also provide novel insights at both theoretical and practical levels.

From a theoretical perspective, by integrating Partial Least Squares Structural Equation Modeling (PLS-SEM), Artificial Neural Networks (ANN), and Necessary Condition Analysis (NCA) within a multi-method framework, this study transcends traditional assumptions of linearity and compensatory relationships. It reveals nonlinear pathways and necessary conditions linking various interaction modalities to emotional, cognitive, and behavioral processes. This methodological integration extends and refines the S–O–R theoretical framework within the context of digital mental health applications. In this renewed formulation, the stimulus (S) is no longer conceived as a singular external input but as an emotionally triggering system composed of multidimensional interaction cues. Similarly, the organism (O) is reconceptualized not as a passive intermediary but as a dynamic field encompassing emotional activation, engagement, and acceptance. This theoretical extension offers a new perspective for understanding users’ self-disclosure behavior: self-disclosure is not merely an expression of willingness but a cognitive and trust-based process grounded in thresholds of emotional arousal and psychological safety.

On the practical level, the findings provide actionable guidance for the affective design of AI systems in mental health contexts. Voice-based and multimodal interactions were found to play decisive roles in enhancing affective engagement and psychological acceptance, suggesting that designers should prioritize the optimization of elements such as vocal tone, facial feedback, and embodied expression to reinforce social presence and trust. Meanwhile, the bottleneck analysis identified the minimal conditions required to trigger behavioral responses, implying that failure to maintain emotional responsiveness or perceived acceptance may render even advanced interaction technologies insufficient to elicit deep self-disclosure. Therefore, future system development should emphasize the dynamic regulation of “emotional thresholds” and the personalization of adaptive mechanisms to enable digital interventions to convey greater human warmth at the algorithmic level.

More importantly, this study suggests that nonlinear affective processes may constitute the foundation for maintaining long-term human–AI relationships. When interactions transcend task-oriented exchanges to achieve emotional resonance, AI chatbots evolve from functional information tools into mediators of companionship and understanding. This finding carries profound implications for the emerging design of “AI companions” intended to deliver sustained psychological support.

### 6.2 Limitation and future research

Although methodological and theoretical novelty was introduced through model development and an integrative analytic strategy, several limitations should be delineated with caution, and clear directions for future research are identified.

First, at the design level, three mental health AI chatbot configurations were operationalized as Wysa (text-based), ChatGPT-5 (voice-based), and Replika (multimodal). While ecological validity was enhanced by conducting the interaction task within real, readily available platforms, “interface modality” was inevitably bundled with platform-level characteristics (e.g., algorithmic capability, degree of anthropomorphism, persona settings, interaction pacing, and interface design style). Accordingly, the observed between-group differences should be interpreted as the joint effects of real-world “system + interaction mode” configurations, rather than as a strict test of the causal effect of interface modality per se. To reduce confounding risk to the extent feasible, task instructions and dialogue themes were held as constant as possible, the number of interaction turns was standardized, subjective experience indicators (e.g., perceived naturalness, clarity, and immersion) were incorporated into the measurement model, and platform familiarity was screened and controlled as a covariate. Nevertheless, the influence of platform differences can be attenuated by these procedures, but it cannot be eliminated. Future research could experimentally manipulate text, voice, and multimodal interfaces within the same underlying model and could further employ a factorial design to disentangle key design dimensions (e.g., modality × anthropomorphic-cue intensity × feedback style). In doing so, “modality effects” could be more precisely separated from “platform-feature effects,” while internal validity is maintained, thereby providing more targeted causal evidence to inform interface design in mental health AI.

Second, with respect to sample composition, participants were primarily young undergraduates, and the proportion of male respondents was relatively high (69.89%). Given that age and gender may shape preferences for emotional expression, thresholds for self-disclosure, perceived privacy boundaries, and subjective definitions of psychological safety, the current sample structure may constrain generalizability to broader populations (e.g., samples with higher proportions of women, more heterogeneous age distributions, or clinical and chronically distressed groups). Therefore, the conclusions should be applied most cautiously to contexts characterized by a relatively young sample with a male majority and prior exposure to mental health applications. In future work, sample diversity could be improved through stratified or quota sampling, and multi-group analyses (e.g., PLS-MGA or multi-group NCA) could be conducted to test whether path relationships and necessary conditions differ across gender and age groups, thereby clarifying boundary conditions regarding for whom and under what circumstances the effects hold.

Third, cross-sectional data were used. Although ANN and NCA were employed to complement mechanistic inference from sufficiency and necessity perspectives, the dynamic evolution of interaction experiences over time could not be examined. During sustained use of mental health AI, emotional arousal, affective engagement, and psychological acceptance may shift from novelty-driven responses to habituation and, potentially, fatigue, which may subsequently influence long-term self-disclosure and intervention effectiveness. Future research could adopt longitudinal designs (e.g., multi-wave measurements), experience sampling methods (ESM), or repeated-interaction assessments in experimental settings to characterize temporal trajectories of psychological mechanisms across configurations and to test differences between short- and long-term effects, as well as conditions under which such effects emerge or change.

In addition, measurement relied primarily on self-report scales and may therefore be affected by social desirability, recall bias, and mood fluctuations—concerns that are especially salient when self-disclosure and mental health are assessed. Under strict ethical and privacy safeguards, objective data sources could be incorporated for triangulation, including behavioral indicators (e.g., conversation duration, pauses and typing rhythms, and number of turns), vocal features (e.g., speech rate, pitch, and pauses), and NLP-derived indices such as textual emotional intensity. Where feasible, physiological signals (e.g., heart rate variability) could also be incorporated to improve construct measurement precision, thereby enabling a more fine-grained depiction of micro-level processes linking emotional change and self-disclosure across configurations.

Finally, although several key paths were cross-validated using SEM, ANN, and NCA, the examination of potential moderating mechanisms was limited. Individual differences (e.g., personality traits, baseline self-disclosure tendency, psychological resilience, and empathy) and contextual factors (e.g., topic sensitivity, perceived privacy risk, and usage motivation) may alter both the formation and magnitude of the psychological mechanisms. Future research could extend the multi-method framework by incorporating multilevel models or Bayesian structural equation models to test cross-level and moderation effects, and qualitative interviews or interaction-content analyses could be incorporated to complement quantitative results. Such efforts would facilitate a deeper understanding of how narratives of being understood and accepted are constructed, how trust and vigilance emerge under different configurations, and how evidence-based personalization and contextualization of mental health AI design can be supported more effectively.

## 7 Conclusion

In summary, this study systematically elucidates the psychological mechanisms and structural patterns through which different interaction modalities facilitate users’ self-disclosure. The findings reveal that text-based, voice-based, and multimodal interactions influence users’ disclosure behaviors through three distinct psychological pathways—emotional arousal, affective engagement, and psychological acceptance—with multimodal interaction exerting the most substantial effect. The multi-method analyses that combined Partial Least Squares Structural Equation Modeling (PLS-SEM), Artificial Neural Network (ANN), and Necessary Condition Analysis (NCA) further demonstrate that the relationships between interaction modalities and psychological mechanisms are neither linear nor compensatory. Instead, they exhibit distinct non-compensatory structures and necessary conditions. Voice and visual cues were found to play pivotal roles in fostering trust, eliciting emotions, and maintaining psychological safety, whereas psychological acceptance emerged as a key factor transforming users’ willingness to express into genuine self-disclosure.

Theoretically, this research extends the explanatory scope of the Stimulus–Organism–Response (S–O–R) framework within the field of affective human–AI interaction. Methodologically, it demonstrates the analytical potential of integrating multiple modeling approaches. Practically, it offers empirical evidence to guide the affective design of AI-based mental health systems. Emotional activation should no longer be conceptualized merely as a system response goal but as a foundational element for understanding human–AI relationships and improving the efficacy of psychological interventions. Looking ahead, as AI technologies continue to advance in embodiment and semantic comprehension, affective intelligence is anticipated to become a foundational component of digital mental health services—shaping not only how individuals communicate but also why they choose to share their inner experiences.

## Data Availability

The original contributions presented in this study are included in this article/Supplementary material, further inquiries can be directed to the corresponding author.
