# Supplementary material for: When AI chatbots understand emotions: exploring the mechanisms of self-disclosure from emotional arousal to psychological acceptance—a hybrid SEM–ANN–NCA analysis across multiple interaction configurations
Source: Front Psychol. 2026 Feb 19;16:1724313. doi: 10.3389/fpsyg.2025.1724313 (PMC12960529; doi:10.3389/fpsyg.2025.1724313)
Supplement: Supplementary file 1 [file Table_1.docx]

Supplementary Material

## Supplementary Figures

| 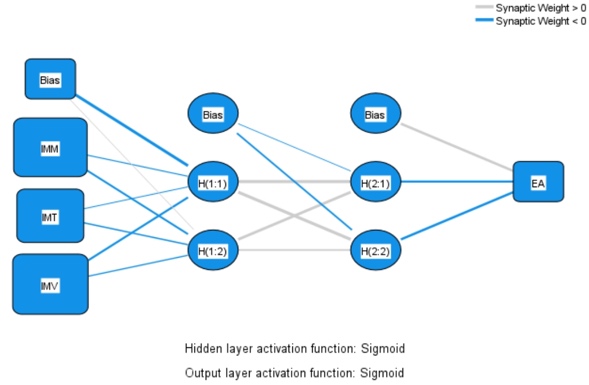 |
| --- |
| *Fig.1* ANN model 1. |
| 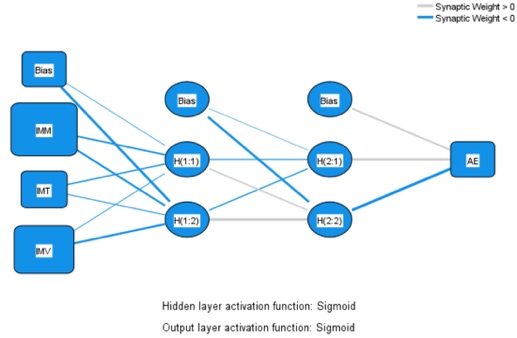 |
| *Fig.2*ANN model 2. |
| 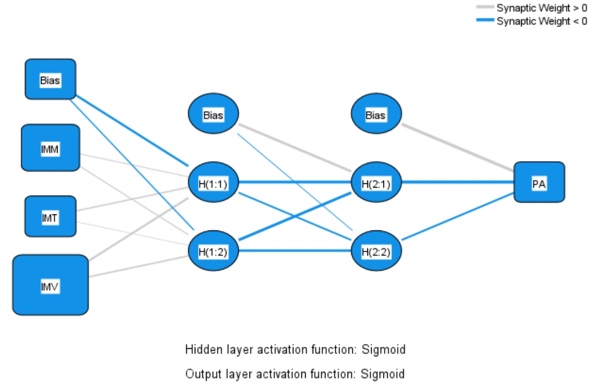 |
| *Fig.3* ANN model 3. |
| 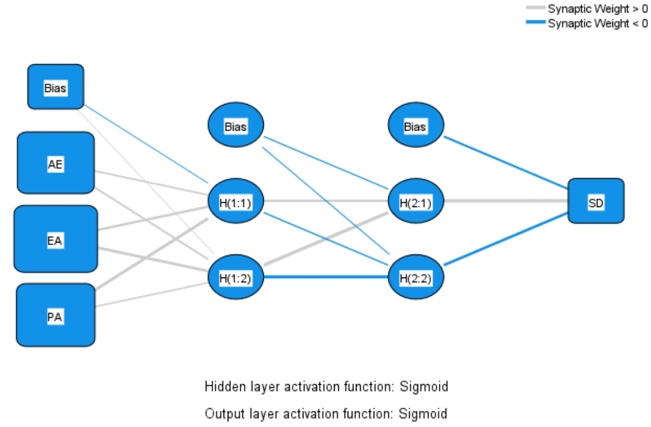 |
| *Fig.4* ANN model 4. |

**Supplementary Figure A1.** Four independent ANN models were developed to examine the nonlinear predictive relationships between interaction modalities (text-based, voice-based, and multimodal interactions) and self-disclosure. Each model used the latent variable scores derived from the PLS-SEM as input nodes. The network architecture consisted of one input layer, one hidden layer, and one output layer. The models were trained using a 10-fold cross-validation approach to ensure robustness and generalizability.

## Supplementary Figures

| 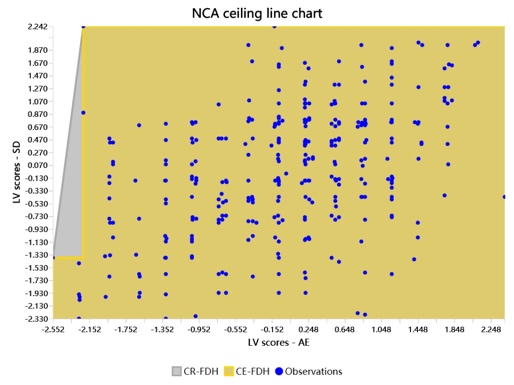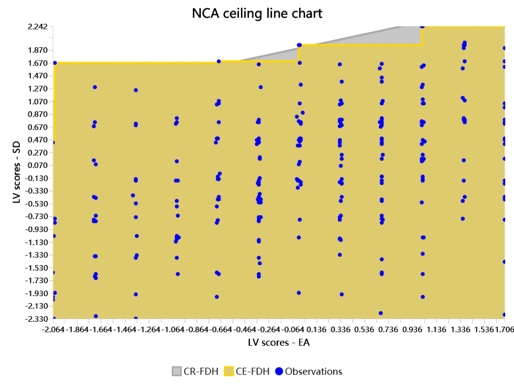 |
| --- |
| *Fig.1* NCA scatter plots for AE against SD. *Fig.2* NCA scatter plots for EA against SD. |
| 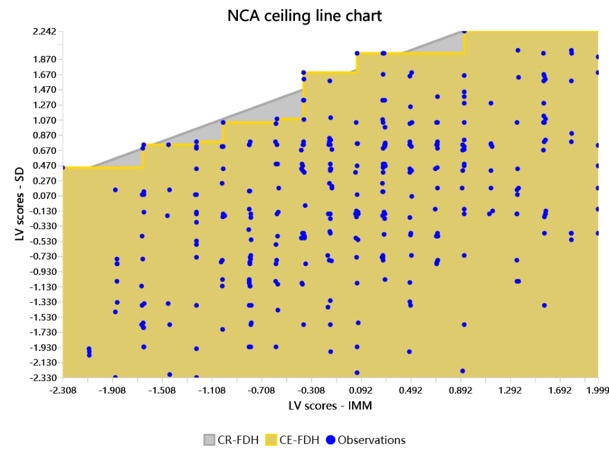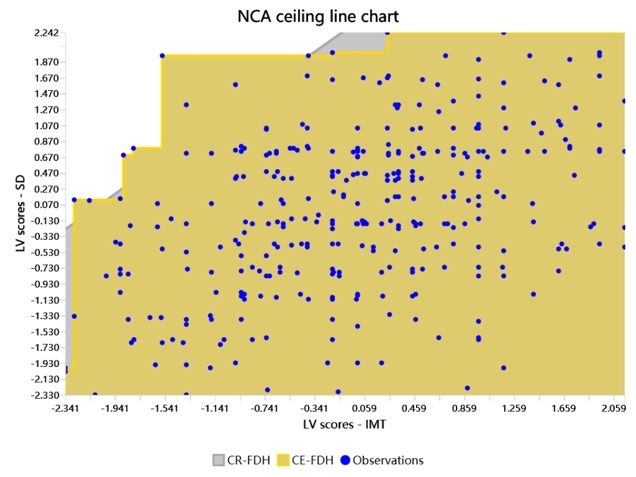 |
| *Fig.3* NCA scatter plots for IMM against SD. *Fig.4* NCA scatter plots for IMT against SD. |
| 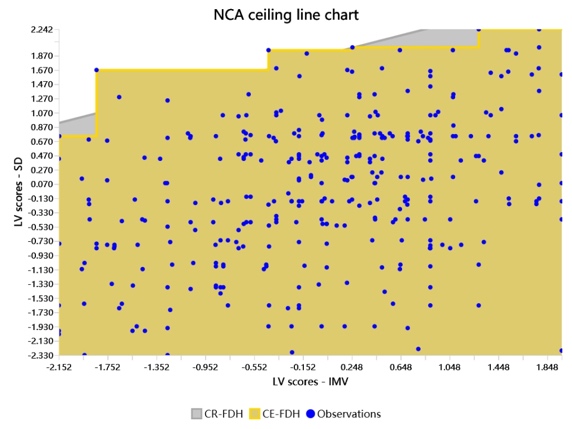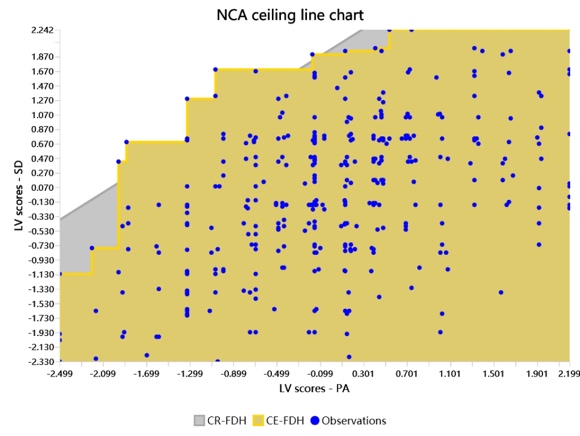 |
| *Fig.5* NCA scatter plots for IMV against SD. *Fig.6* NCA scatter plots for PA against SD. |
| 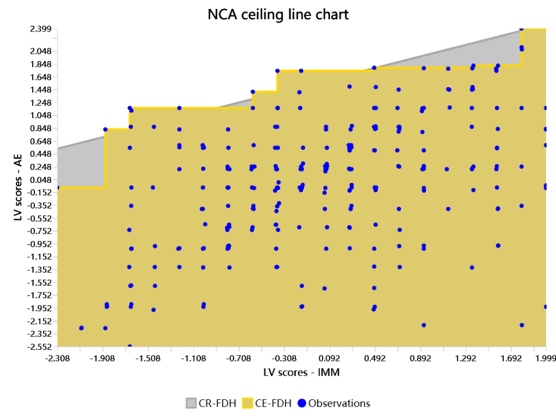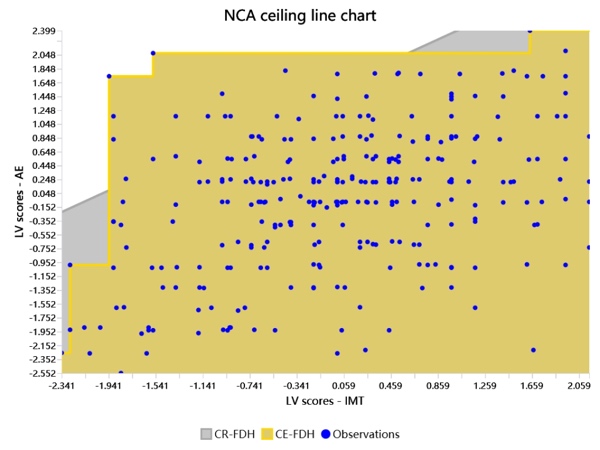 |
| *Fig.7* NCA scatter plots for IMM against AE. *Fig.8* NCA scatter plots for AE against IMT. |
| 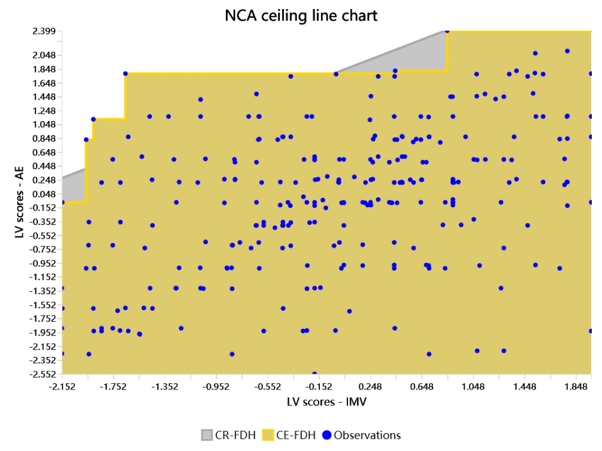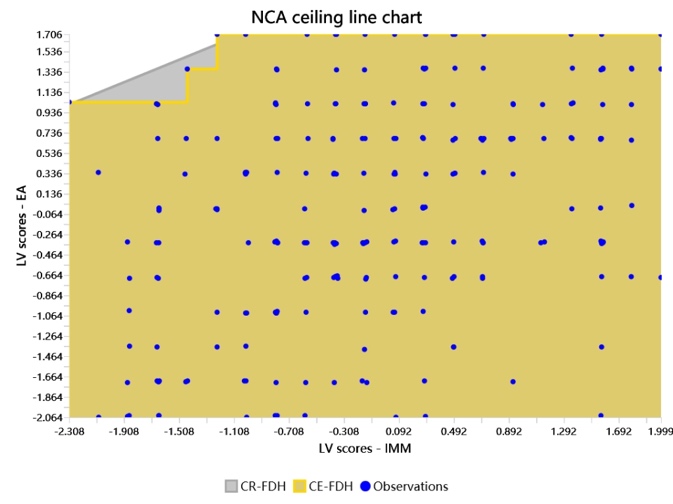 |
| *Fig.9* NCA scatter plots for IMV against AE. *Fig.10*NCA scatter plots for IMM against EA. |
|  |
| 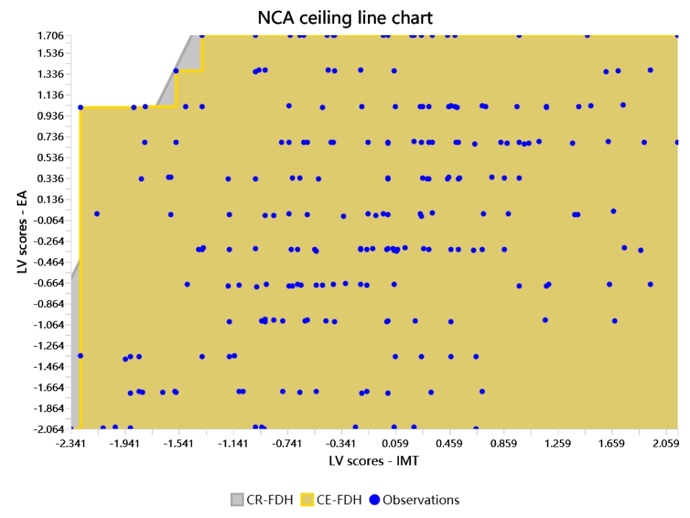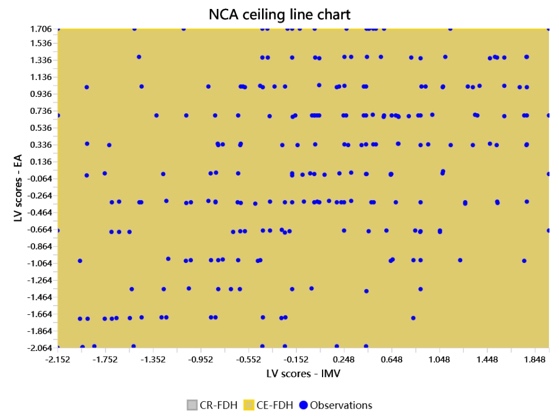 |
| *Fig.11* NCA scatter plots for IMT against EA. *Fig.12*NCA scatter plots for IMV against EA. |
| 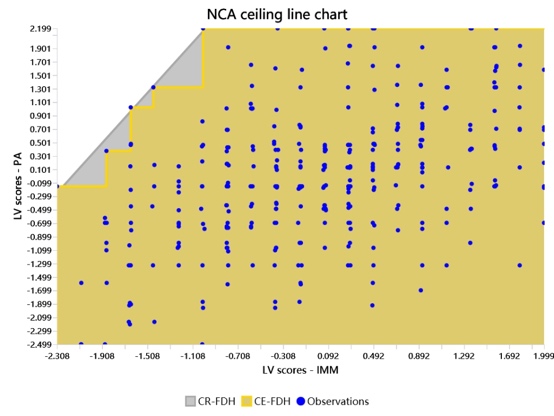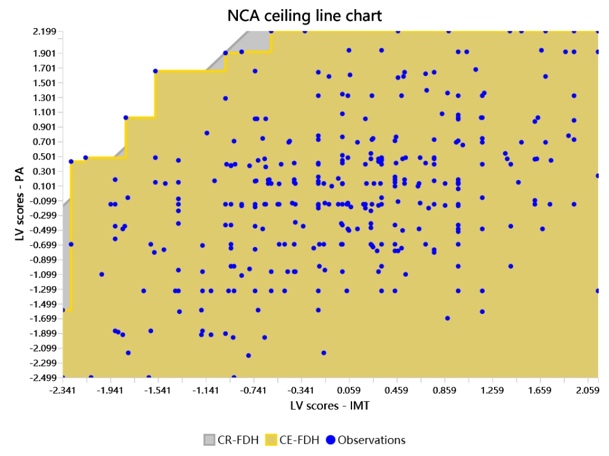 |
| *Fig.13*NCA scatter plots for IMM against PA. *Fig.14* NCA scatter plots for IMT against PA. |
|  |
| 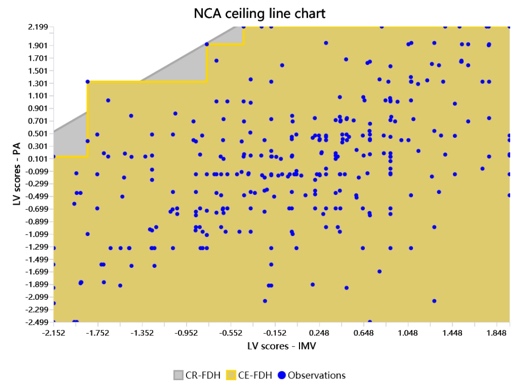 |
| *Fig.15* NCA scatter plots for IMV against PA. |

**Supplementary Figure A2.** Scatterplots of the Necessary Condition Analysis (NCA) results showing the ceiling lines (CR-FDH) and bottleneck levels for the three key psychological mechanisms: emotional arousal, affective engagement, and psychological acceptance. These plots demonstrate the necessary condition effects for self-disclosure in AI-driven interactions.
